# The Development of the Human Female Reproductive Tract: Part 2—Vagina

**DOI:** 10.1002/ca.70015

**Published:** 2025-08-24

**Authors:** Cindy J. M. Hülsman, S. Eleonore Köhler, Gabriela Morosan‐Puopolo, Jill P. J. M. Hikspoors, Wouter H. Lamers

**Affiliations:** ^1^ Department of Anatomy & Embryology Maastricht University Maastricht the Netherlands; ^2^ Department of Anatomy & Molecular Embryology Ruhr University Bochum Bochum Germany

**Keywords:** mesonephric (Wolffian) duct, paramesonephric (Müllerian) duct, urogenital sinus, uterovaginal canal, vagina

## Abstract

Urogenital epithelium replaces the original paramesonephric (Müllerian) epithelium in the human fetal vagina. We re‐investigated this cell replacement histologically and with three‐dimensional reconstructions. In the lesser pelvis, both Müllerian ducts fuse to form the uterovaginal canal. Its large caudal “head” is flanked by the locally widened Wolffian ducts. In the 9th week, the urogenital epithelium that covers the Müllerian tubercle differentiates into small, dense‐staining basal cells and columnar “clear cells” apically. Mesonephric (Wolffian‐duct) outlet epithelium in contact with clear cells degenerates and is replaced by clear‐cell epithelium. Meanwhile, the small cells of the urogenital‐sinus epithelium form a funnel that physically contacts the underlying uterovaginal head, locally breaks down its basement membrane, and establishes a bridgehead. Here, extensive cell mixing of urogenital and Müllerian cells produces a “transformational” epithelium with regressing Müllerian and healthy urogenital cells as components. After spreading throughout the vaginal epithelium, the urogenital cells near their incursion site under the Wolffian‐duct outlets begin to produce the bilateral “vaginal plates.” Its cells surround the transformational epithelium as a deflated double‐layered cell plate. Where the vaginal plates contact the transformational epithelium, the epithelial cell layer thickens, its surface becomes rugged, and large regressive cells become numerous. The number of regressive cells drops precipitously in the adjacent vaginal plates, implying that this band of cells functions as a “purging zone.” Once the purging process reaches the midline, the vaginal epithelium consists of urogenital cells only. Their arrangement as a deflated double‐layered cell plate temporally occludes the vaginal lumen.

**Abbreviations**
B: bladder
C: cervix
Cau: caudal
CC: clear cells
Cr: cranial
D: dorsal
DM: dense mesenchyme
L: left
MD: Müllerian duct
P: pubic bone
PZ: purging zone
R: right
SE: sinusal epithelium
TE: transformational epithelium
U: urethra
UGS: urogenital sinus
Ur: ureter
UVC: uterovaginal canal
V: ventral
Va: vagina
Vest: vaginal vestibule
VP: vaginal plate
W: weeks
WD: Wolffian duct

## 1 Introduction

Since Robboy and colleagues phenotyped the urogenital organs of human embryos and fetuses with antisera against PAX2 (staining the Müllerian epithelium) and FOXA1 (staining the urogenital epithelium) (Robboy et al. 2017), the discussion about the replacement of Müllerian epithelium by urogenital epithelium in human fetuses seems largely settled. Robboy et al. do not, however, offer a model that predicts the movement of the urogenital cells into the Müllerian epithelium of the vagina and the subsequent selective elimination of non‐Müllerian cells that can be experimentally tested in animal models. The first part of this study focuses on the development of the uterine tubes and the uterus (Hülsman, Köhler, et al. 2025a), while this second part describes the early formation and differentiation of the vagina and aims to provide such a testable model.

The fate of the paramesonephric (Müllerian) epithelium in the vaginal part of the uterovaginal canal and its apparent replacement by external epithelial cells was already a focus of discussion well over a century ago. Tourneux and Legay reported a contribution of mesonephric (Wolffian) duct epithelium to the lower part of the definitive uterovaginal canal (Tourneux and Legay 1884). Nagel produced a remarkably accurate account of the development of the Müllerian duct (Nagel 1891), in which he reported the persisting lack of fusion of left and right Müllerian ducts near the Müllerian tubercle and, following Tourneux and Legay's account, a stratified epithelium in the caudal as opposed to a simple columnar epithelium in the cranial part of the uterovaginal canal of 9‐week‐old fetuses. He concluded, nonetheless, that the vaginal epithelium was and remained of Müllerian origin. A century ago, Bloomfield and Frazer classified the respective opinions in a perhaps overly schematic way (Bloomfield and Frazer 1927): (1) the vaginal epithelium derives in its entirety from the Müllerian epithelium, with Nagel (1891), Felix (1912), Bloomfield and Frazer (1927), Hunter (1930), von Lippmann (1939), and Matejka (1959) as prominent supporters; (2) the vaginal epithelium forms in part (Tourneux and Legay 1884; Mijsberg 1924), or in its entirety (Kempermann 1931) by incorporating cells of the dilated distal ends of the Wolffian ducts; or (3) the vaginal epithelium derives partly (its lower segment; Bolk 1907; Koff 1933) or entirely (Vilas 1932; Kempermann 1935; Meyer 1934, 1936, 1937, 1938; Bulmer 1957) from the urogenital epithelium.

Vaginal development was also studied in mouse models. Drews investigated androgen‐insensitive mice and concluded that the vagina develops by down‐growth of Wolffian and Müllerian ducts along the sinus ridges. Due to a low expression of the androgen receptor, the caudal ends of the Wolffian ducts regress so that the definitive vagina is formed from the Müllerian ducts (Drews 2007). More recently, Kurita (2010) genetically labeled the respective epithelial cells in mice and traced their fate in the Müllerian ducts. In neonates, the cranial portion of the epithelium of the fused Müllerian ducts was of Müllerian origin and the caudal portion of urogenital origin. During its further downgrowth in the first postnatal week, however, the cranial (Müllerian) portion of the vagina continued to growth caudally, whereas the caudal (urogenital) portion did not, so that the entire vagina became eventually lined with epithelium of Müllerian origin (Kurita 2010). The place of entrance of the vagina into the urogenital sinus is subsequently remodeled by lateral growth prenatally and longitudinal growth postnatally (Harada and Akita 2023). These relatively recent analyses in mice support the Müllerian epithelial origin of the vaginal epithelium.

Key structures in these diverging opinions are the caudal ends of the Wolffian ducts, which are very wide relative to their more upstream parts and are, therefore, often described as Wolffian bulbs (or Höcker; Mijsberg 1924), but also as “sinus bulbs” (Vilas 1932), “Müllerian bulbs” (Bloomfield and Frazer 1927), or “sinovaginal bulbs” (Koff 1933). In the models that only allow a limited contribution of Wolffian or urogenital epithelium to the vaginal epithelium, this material is assumed to derive from these bulbs. According to Drews (Drews 2007; Mauch et al. 1985), Koff inadvertently attributed a urogenital rather than a Wolffian‐duct origin to the epithelium of his “sinovaginal bulbs,” because he apparently thought that they were large evaginations of the dorsal wall of the urogenital sinus.

The account of Koff (1933) is at present the most ubiquitous version of vaginal epithelial development in textbooks of human embryology, even though Koff provides only an equivocal description of the sinovaginal bulbs (“bilateral bulb‐shaped evaginations of the posterior (dorsal) wall of the urogenital sinus in this region”; p. 62) and does not include a single histological section of this key structure of his model. Moreover, Koff provides only a single contour (his Plate 2D) and a scanty definition of the vaginal plates (“the solid portion of the sinovaginal bulbs that coalesces with the tip of the uterovaginal canal,” p. 72), another mainstay of his model.

A partial or complete replacement of Müllerian epithelial cells by epithelial cells of urogenital or Wolffian‐duct origin should, in all likelihood, be visible histologically. As far as we know, however, such observations have not been made. Our main findings are that the columnar Müllerian epithelium is invaded between 8.5 and 11 weeks of development by small urogenital epithelial cells without losing its epithelial appearance. Subsequently, the Müllerian cells are selectively eliminated between 12 and 16 weeks in a process that progresses from caudal to cranial and from lateral to medial through the vagina.

## 2 Materials and Methods

The materials and methods used were identical to those described in the accompanying study (Hülsman, Köhler, et al. 2025a). The fetuses that were studied in detail to describe the early development of the vaginal epithelium are listed in Table 1. The interactive 3D‐PDFs available as supplemental information in part‐1 can also be used to understand the complex local topographies of structures described in the text or illustrated in the figures. We do not separately refer to these 3D‐PDFs in the main text to avoid crowding the text with similar words. In addition to the 3D‐PDFs included in part‐1, three further 3D‐PDFs of the urogenital region of 8.5‐week (Figure S5), ~14 weeks (Figure S6), and ~20 weeks (Figure S7) fetuses are available.

**TABLE 1:** Overview of human embryos and fetuses used in the study.

Weeks	Number	CRL^a^ (mm)	Plane	Source
8	S48	40	Transverse	LUMC
8	S4141	35	Transverse	AMC
8	S88	24	Sagittal	Nijmegen
8.5	EYO295	32	Sagittal	Bochum
9	S89	37	Transverse	LUMC
10‐early	S4908	50	Transverse	AMC
10‐late	S1744	60	Transverse	LUMC
11	S1743	80	Sagittal	LUMC
12	S2383	105	Transverse	LUMC
13	S2212	110	Sagittal	LUMC
14	ME29/534E	105	Coronal	Bochum
15	S2392	130	Transverse	LUMC
20	S2290	200	Sagittal	LUMC

^a^ Crown‐rump length.

## 3 Results

### 3.1 List of abbreviations and color codes

**Table:** 

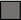	B	bladder
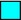	C	cervix
	Cau	caudal
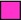	CC	clear cells
	Cr	cranial
	D	dorsal
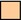	DM	dense mesenchyme
	L	left
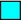	MD	Müllerian duct
	P	pubic bone
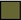	PZ	purging zone
	R	right
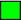	SE	sinusal epithelium
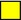	TE	transformational epithelium
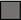	U	urethra
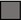	UGS	urogenital sinus
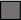	Ur	ureter
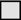	UVC	uterovaginal canal
	V	ventral
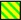	Va	vagina
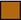	Vest	vaginal vestibule
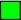	VP	vaginal plate
	W	weeks
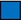	WD	Wolffian duct

### 3.2 The Establishment of a Urogenital Epithelial Bridgehead in the Head of the Müllerian Duct

**FIGURE 1:**
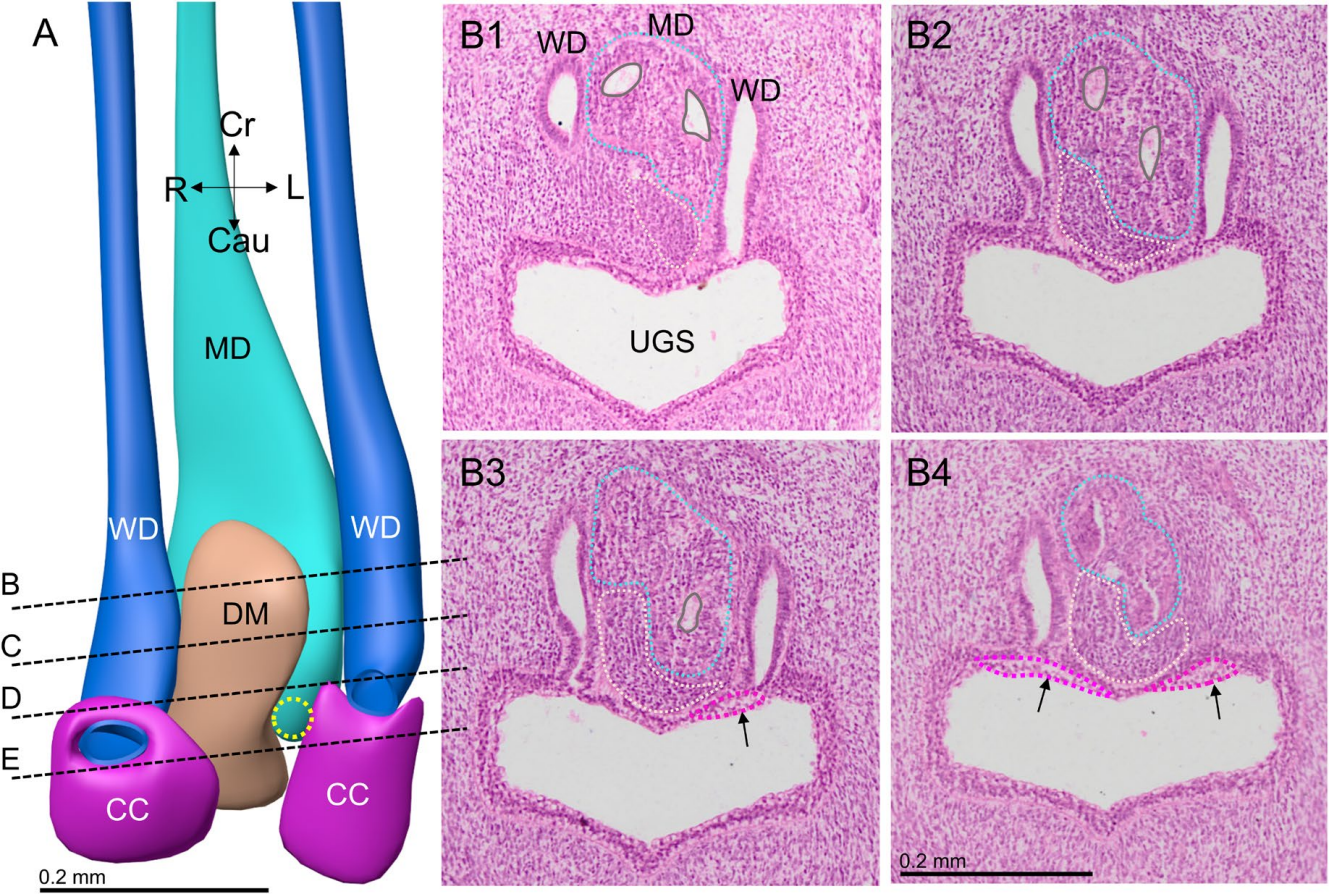
Asymmetric growth in the caudal non‐fused part of the uterovaginal canal at 8 weeks (embryo s48). Panel (A) shows the epithelial contours of the uterovaginal canal (cyan), the Wolffian ducts (blue), the mesenchymal wedge in the Müllerian tubercle (peach) and the sinusoidal clear cells (magenta). The dense mesenchymal wedge (dotted peach contours in panels (B1–4); positions shown in panel (A)) has formed asymmetrically between the epithelia of the uterovaginal head and that of the urogenital sinus, so that only the left‐sided part of the uterovaginal head (dotted cyan contours) can contact the urogenital sinus (at the dotted yellow circle in panel (A)). Around the outlet of the Wolffian ducts into the urogenital sinus, so‐called “clear cells” rather than Wolffian‐duct cells now line the Wolffian‐duct lumen (magenta cuffs in panel (A) and contours in panels (B1–4)). Cau: caudal; CC: clear cells; Cr: cranial; DM: dense mesenchyme; L: left; MD: Müllerian duct; UGS: urogenital sinus; WD: Wolffian duct. Bars: 0.2 mm.

**FIGURE 2:**
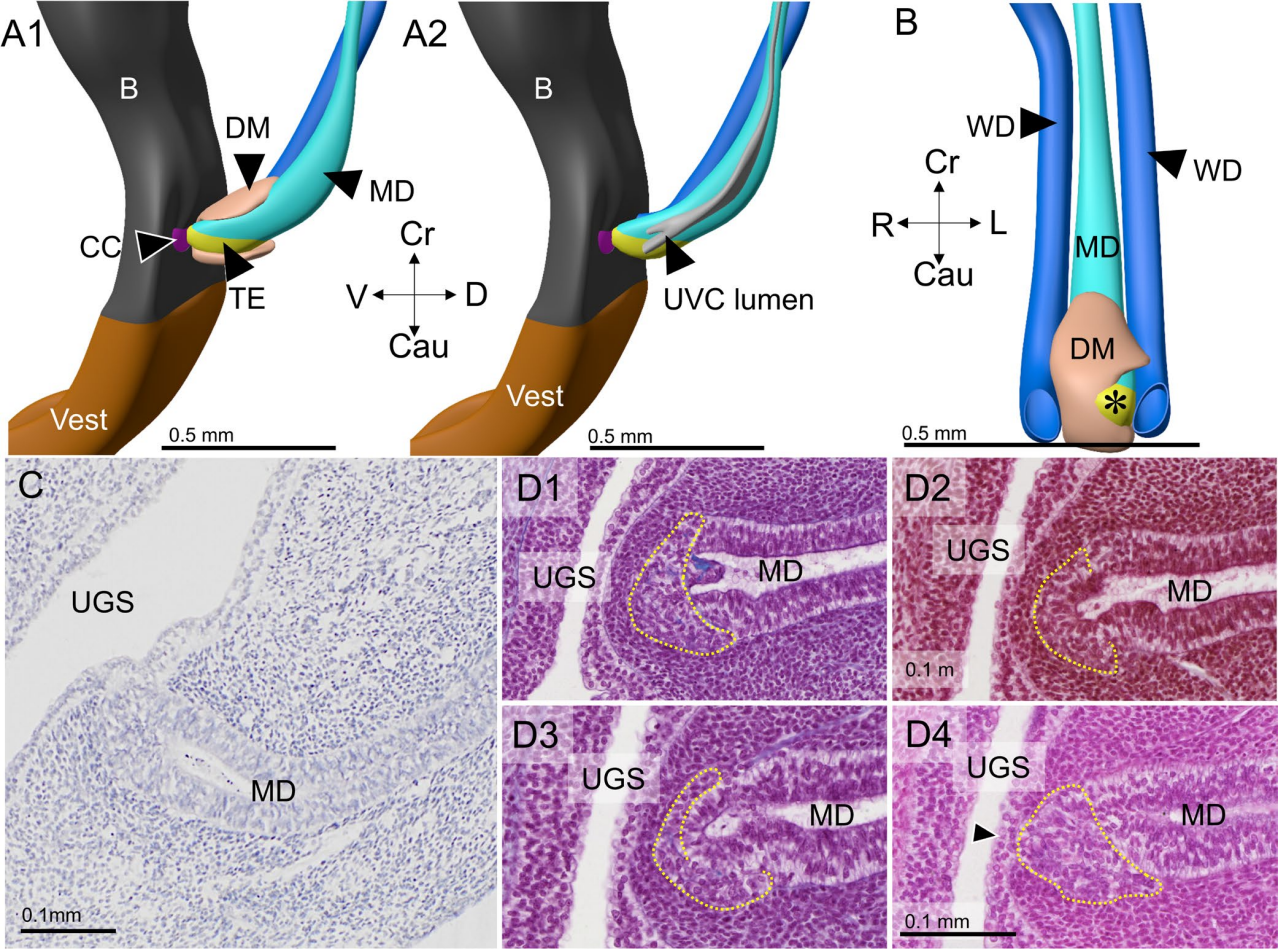
Establishing a direct contact between the Müllerian‐duct and urogenital epithelium in 8–8.5‐week‐old embryos (s88 and EYO295). Panels (A1) and (A2) show 3D images of the epithelium of the Müllerian (cyan) and Wolffian ducts (blue) of an 8.5‐week fetus. The bladder and the pelvic part of the urethra are coded gray, and the phallic part (vestibule) brown. In panel (A1), the left Wolffian duct is removed to expose the left side of the Müllerian duct. Only this left side can contact the urogenital‐sinus epithelium. In panel (A2), the Müllerian epithelium is rendered transparent to reveal the bifid end of the uterovaginal canal (light gray). Panel (B) shows a frontal view of the same area to visualize the contact site of the top of the left‐sided part of the uterovaginal canal (asterisk) and the urogenital sinus. Panel (C) shows a sagittal section through this contact site in the 8‐week‐old embryo (s88), and panels (D1–4) that in the 8.5‐week‐old embryo (EYO295). Dorsally (right side of the images), the epithelial arrangement of the Müllerian cells is standard, whereas cell mixing of urogenital and Müllerian‐duct epithelial cells (yellow dashed contours) has started at the apex of the uterovaginal canal (panel (D1), contours were partially deleted in panels (D2) and (D3) to show interrupted basement membrane). Note that the mixing zone on the head of the uterovaginal canal (panel (D1)) is fed by urogenital cells that lie ~300 μm leftward (panel (C4)). B: bladder; Cau: caudal; CC: clear cells; Cr: cranial; D: dorsal; DM: dense mesenchyme; MD: Müllerian duct; TE: transformational epithelium; UGS: urogenital sinus; UVC: uterovaginal canal; Vest: vaginal vestibule; WD: Wolffian duct. Bars: 0.5 mm for reconstructions and 0.1 mm for sections.

**FIGURE 3:**
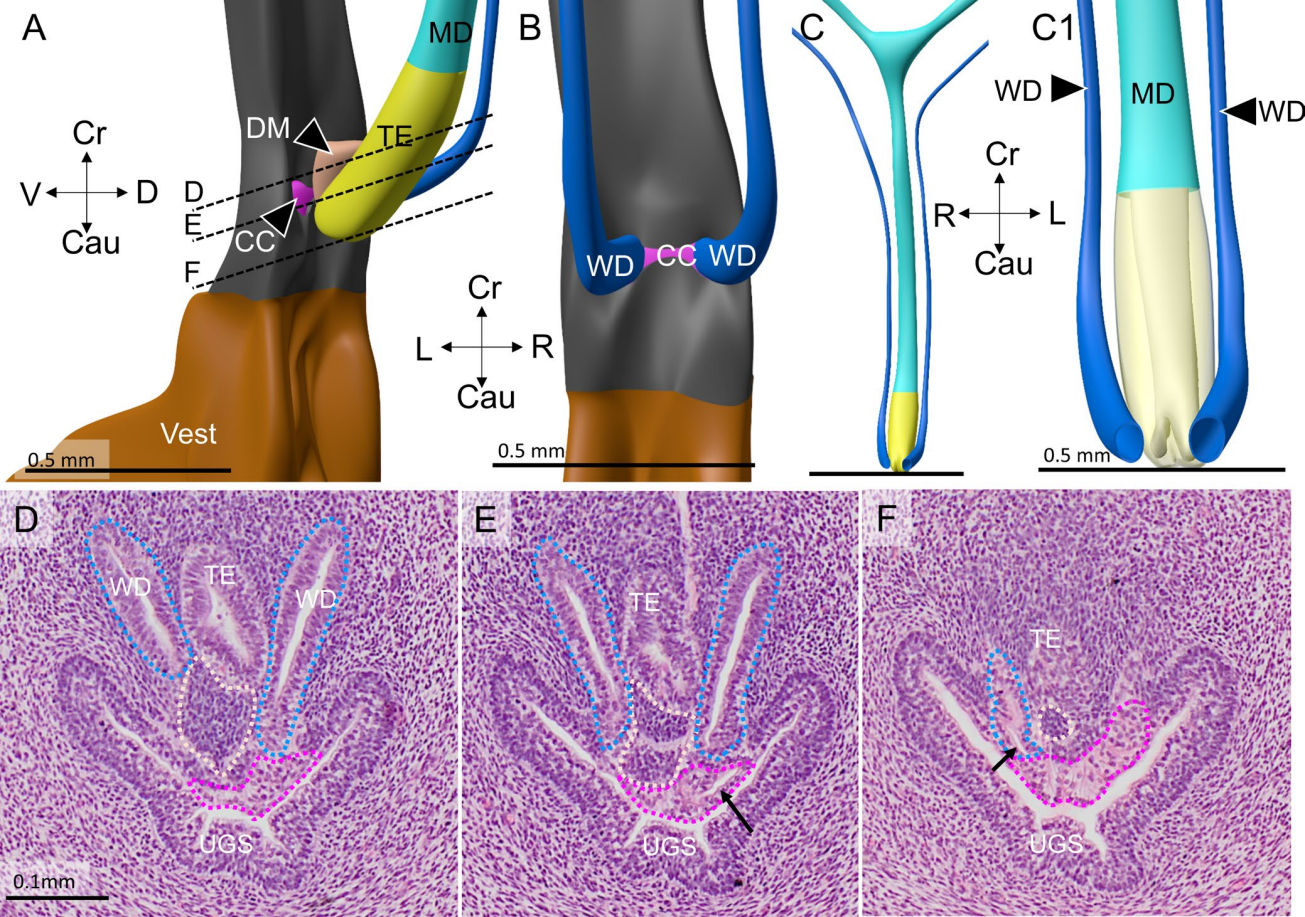
Urogenital epithelial cells infiltrate into the caudal end of the uterovaginal canal (fetus s4908). Panels (A) and (B) show a left dorsolateral and a dorsal view, respectively, of the uterovaginal canal, Wolffian ducts, and urogenital sinus at ~10 weeks of development, while panels (C) and (C1) (magnification) show a ventral view of the uterovaginal canal and the Wolffian ducts of this fetus. Panel (C1) reveals the bifid caudal end of the uterovaginal canal. Dense mesenchyme (panels (A) and (D–F)) has inserted itself mainly between the right‐sided uterovaginal canal and the urogenital sinus, allowing the urogenital epithelium to contact the uterovaginal canal on the left side only. Clear cells (delineated by the magenta‐dotted line) cover the entire Müllerian tubercle (panels (D–F); positions shown as dashed lines in panel (A)), and surround the outlets of the Wolffian ducts (arrows in panels (E) and (F)). On these transverse sections, the urogenital lumen resembles a butterfly, with its large wings dorsolaterally flanking the Wolffian ducts and its small wings ventrolaterally. The uterovaginal canal loses its basement membrane only where it contacts urogenital sinus epithelium and the dense mesenchymal wedge (panel (D)). B: bladder; Cau: caudal; CC: clear cells; Cr: cranial; D: dorsal; DM: dense mesenchyme; L: left; MD: Müllerian duct; R: right; TE: transformational epithelium; UGS: urogenital sinus; V: ventral; Vest: vaginal vestibule; WD: Wolffian duct. Bars: 0.5 mm (top row), and 0.1 mm (bottom row).

**FIGURE 4:**
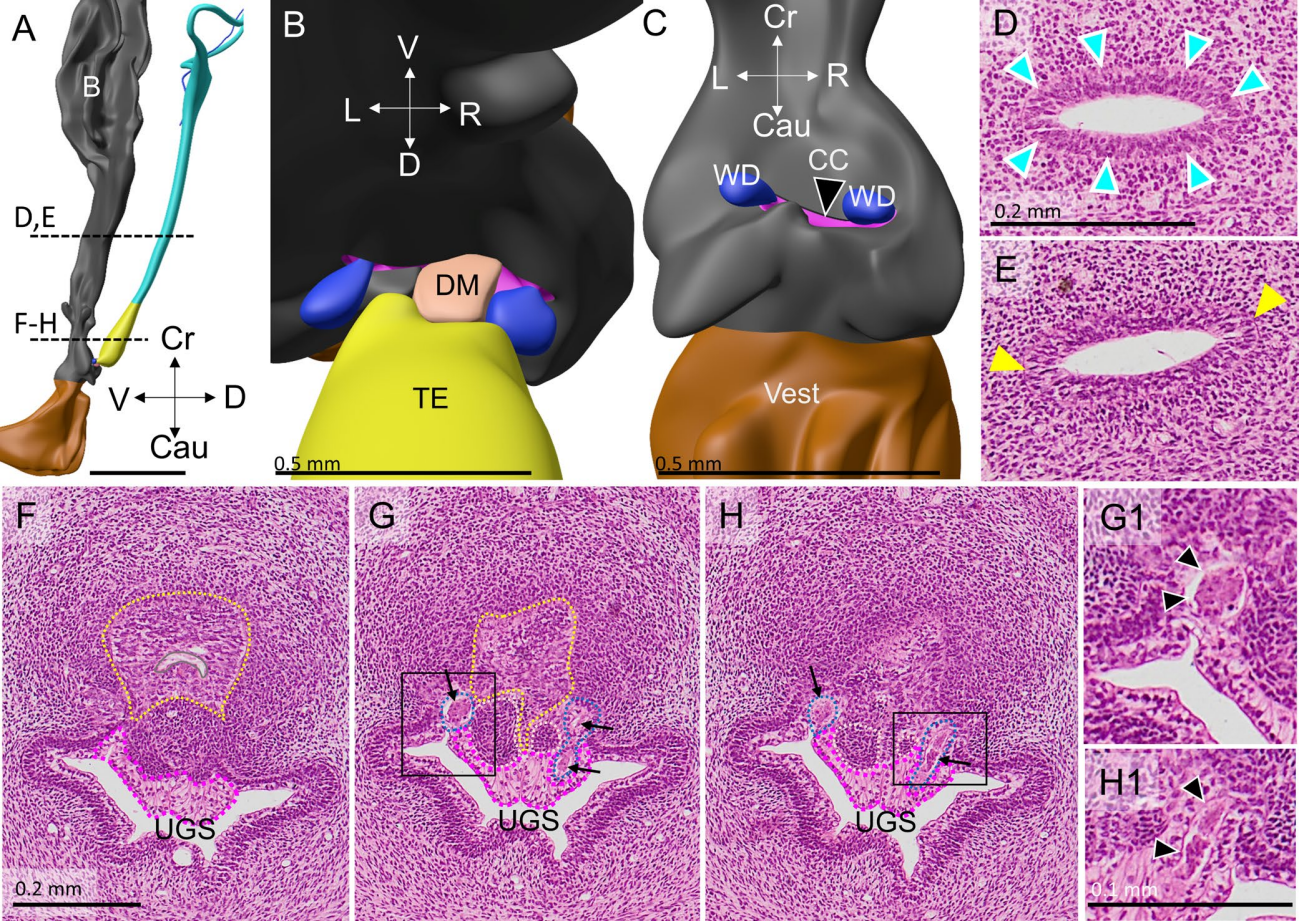
Continued infiltration of the caudal uterovaginal epithelium by urogenital epithelial cells (fetus s1744). Panel (A) shows a left‐lateral image at the end of the 10th week. Panels (B) and (C) show caudo‐ and craniodorsal views of the Müllerian tubercle, respectively, to reveal the asymmetric contact of the left fork of the uterovaginal epithelium (yellow) with the urogenital epithelium (gray). The asymmetric dense mesenchymal wedge is still a right‐sided structure that is ventrally bordered by clear cells (CC). The Wolffian ducts (WD) delimit the Müllerian tubercle laterally. Note that the caudal vagina (yellow) is not shown in panel (C). Panels (D) and (E) show the uterovaginal canal near the uterovaginal junction: Panel (D) shows the typical histological arrangement of cells and basement membrane (cyan arrowheads) of the Müllerian epithelium, whereas the denser packing of nuclei and the locally interrupted basement membrane in the slightly more caudal section (E) (yellow) demonstrates infiltration by typically small urogenital cells. The positions of panels (D)–(H) is shown as dashed lines in panel (A). Panels (F)–(H) show the clear cells (dashed magenta contours) are most prominent on the Müllerian tubercle, with a much thinner layer covering the remaining surface of the urogenital sinus. The butterfly configuration shown in Figure 3 is still recognizable. Note the dying epithelial cells in the Wolffian ducts near their outlets (arrows; magnifications in panels (G1) and (H1)), where they are bordered by clear cells (magenta‐dotted line in panels (G) and (H)). Also note that the uterovaginal cells of the large head (yellow outlines in panels (F) and (G), but not shown in panel (H) to allow inspection of the basement membrane) locally have a high nuclear density and miss a basement membrane except at their left and right sides (*cf*. panels (D) and (E)). B: bladder; Cau: caudal; CC: clear cells; Cr: cranial; D: dorsal; DM: dense mesenchyme; L: left; R: right; TE: transformational epithelium; UGS: urogenital sinus; V: ventral; Vest: vaginal vestibule; WD: Wolffian duct. Bars: 0.5 mm for the reconstructions, 0.2 mm for the sections, and 0.1 mm for the magnifications.

**FIGURE 5:**
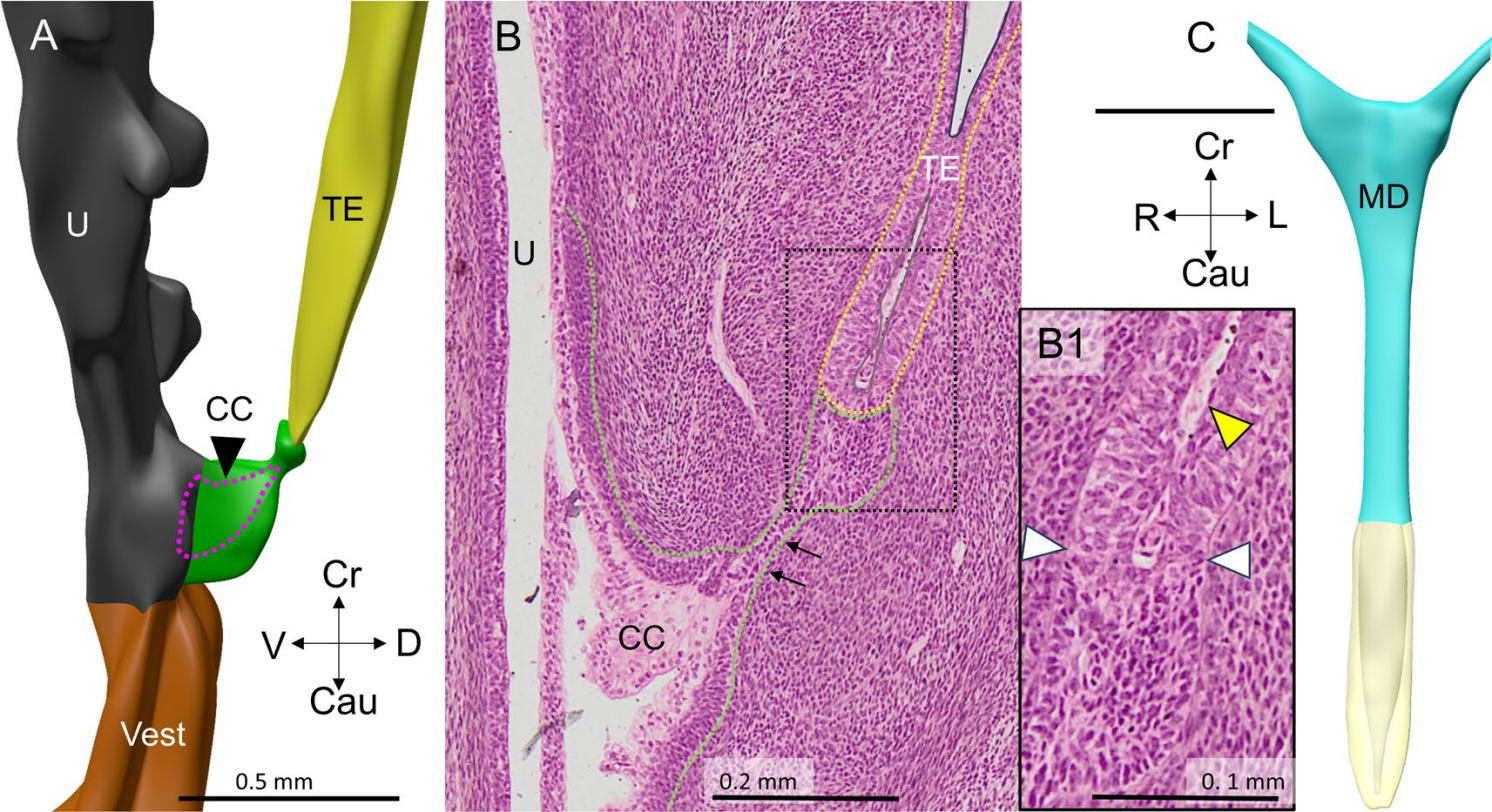
Inversion of the Müllerian tubercle into a funnel and completion of the vaginal epithelial infiltration by urogenital epithelial cells (fetus s1743). Panels (A) and (C) show a left‐lateral and a frontal view of the uterovaginal canal (yellow; transparent in panel (C)) of an ~11‐week‐old fetus. Panel (B) is a median section that shows that the bulging of its surrounding tissues changes the Müllerian hill into a funnel. The funnel (dashed bright‐green contour) is covered by a basal layer of small, densely staining urogenital epithelial cells and large clear cells at its surface (CC). Just before reaching the head, the sinusal‐cell column widens into an intumescence, with basal cells in its periphery and randomly oriented cells in the center. The connection between the funnel and the intumescence is only 2‐cells thick (small black arrows in panel (B)). The basement membrane is absent locally at many places and totally absent at the contact site with the uterovaginal canal (white arrowheads in panel (B1)). The uterovaginal canal is lined by transformational epithelium (dashed yellow contour in panel (B)), which contains a mix of short and tall cells, but remains identifiable by its central lumen (yellow arrowhead in panel (B1)) and its locally present basement membrane. Cau: caudal; CC: clear cells; Cr: cranial; D: dorsal; L: left; MD: Müllerian duct; R: right; TE: transformational epithelium; U: urethra; V: ventral; Vest: vaginal vestibule. Bars: 0.5 mm for reconstructions, 0.2 mm for the section and 0.1 mm for the magnification.

**FIGURE 6:**
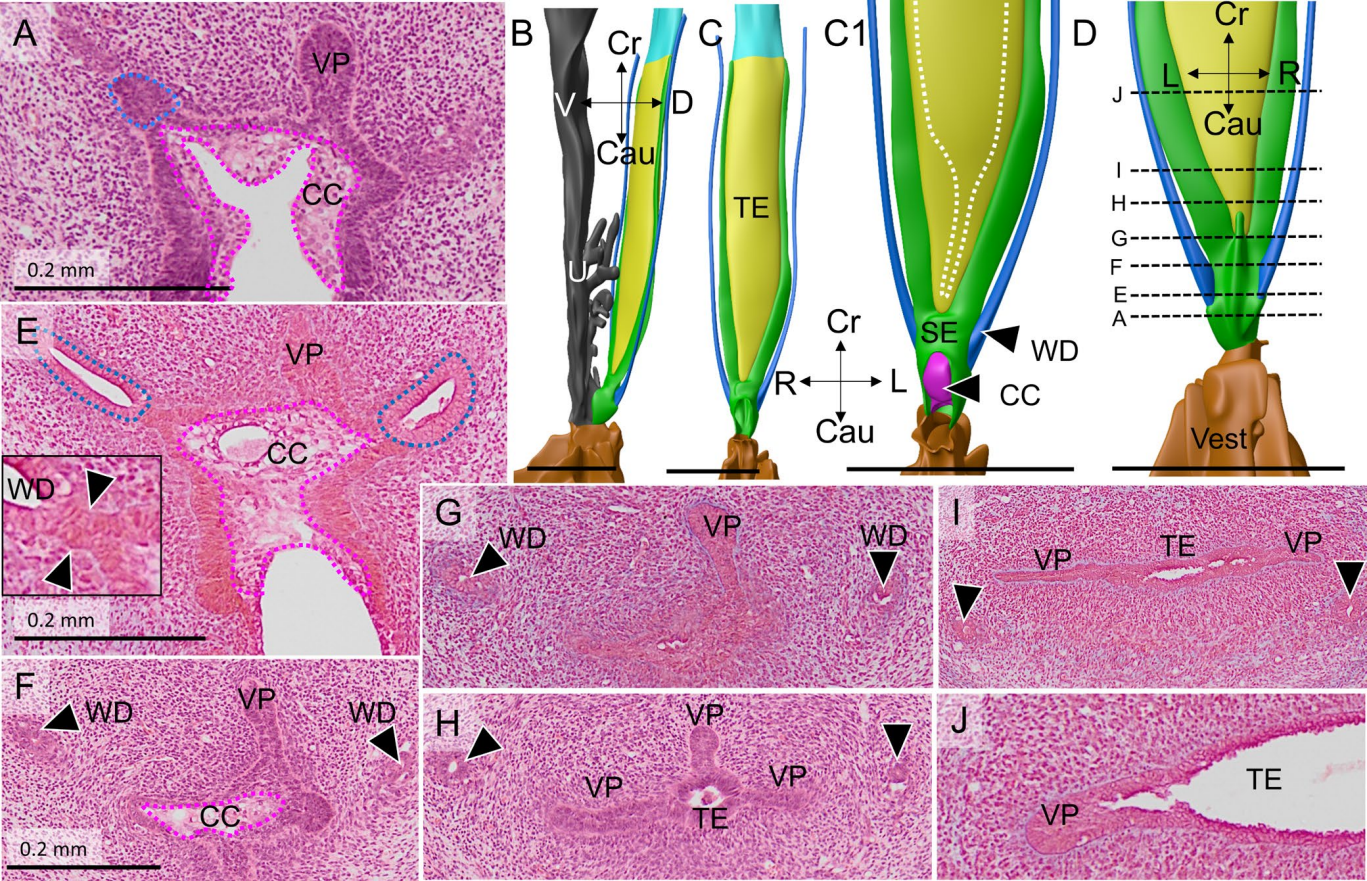
The early development of the vaginal plates (fetus s2383). Panels (B–D) show left‐lateral (B), ventral (C and C1) and dorsal (D) views of the vaginal part of the uterovaginal canal. Panels (A) and (E–G) show the funnel of the urogenital sinus changing into the head of the uterovaginal canal, with a wide lumen and tall epithelium (panel (H)), while panel (J) shows that the uterovaginal lumen widens substantially in its cranial part. Here, the vaginal plates form laterally. Panels (A), and (E–J) show transverse sections of the genital cord at the levels indicated in panel (D). The sinusal funnel (panels (A), (E–G)) is lined by basal urogenital epithelium, which is covered by clear cells. In panels (A) and (E), the Wolffian ducts (dashed blue lines) enter the funnel, while panels (F–J) show the more cranial course of these ducts (black arrowheads). In panels (A), (G), and (H) the early formation of the mediodorsal (short) vaginal plate (reconstructed in green as a median ridge in panel (D)) is seen, while in panels (H–J) the lateral vaginal plates (also coded in green) form just caudal to the outlets of the Wolffian ducts. Note the well‐developed basement membrane of the vaginal plates and the tête‐à‐tête arrangement of their epithelial cells (panels (H–J)). Cau: caudal; CC: clear cells; Cr: cranial; D: dorsal; L: left; R: right; SE: sinusal epithelium; TE: transformational epithelium; U: urethra; V: ventral; Vest: vaginal vestibule; WD: Wolffian duct. Bars: 1 mm for the reconstructions and 0.2 mm for the sections.

**FIGURE 7:**
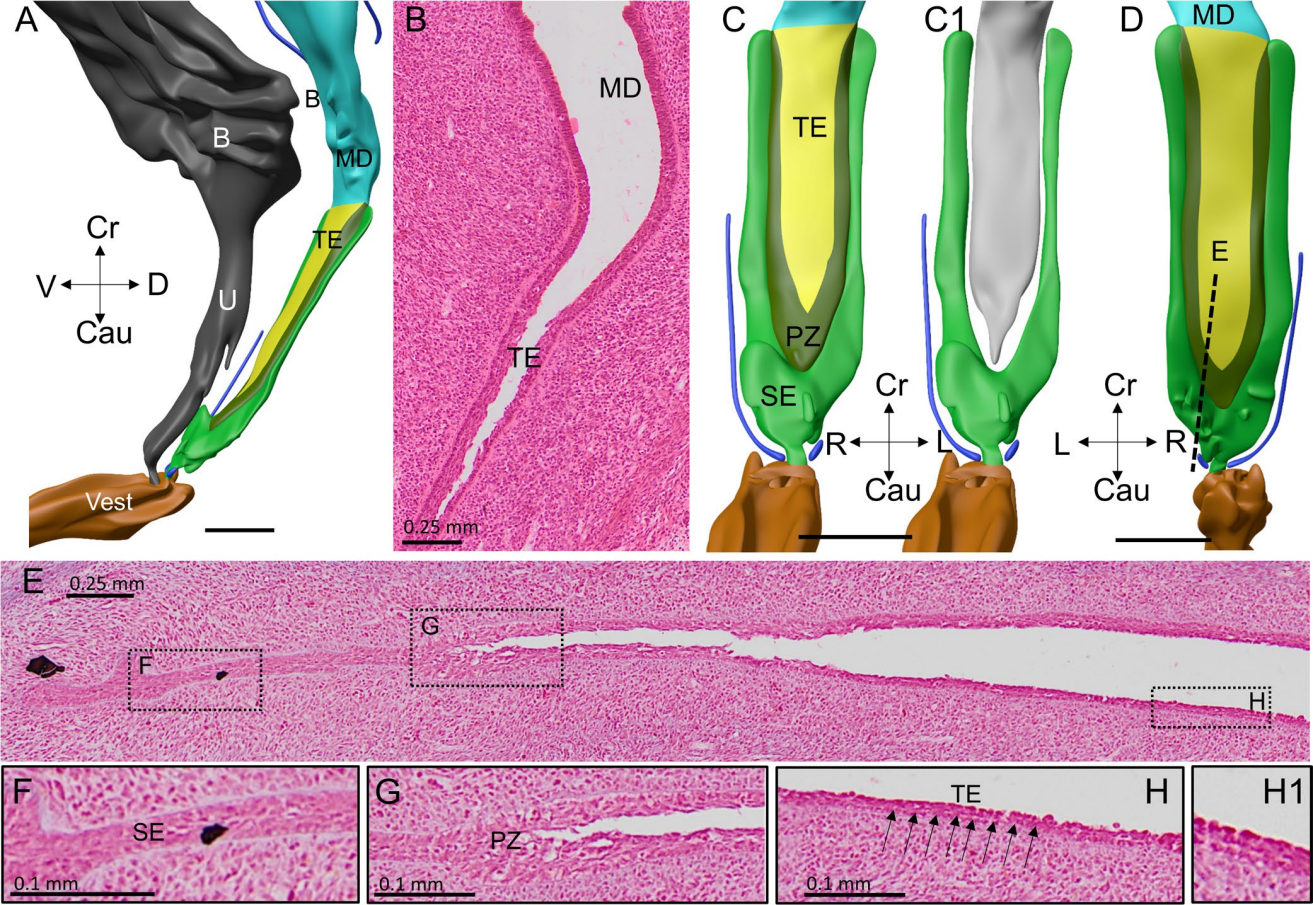
The histology and topography of the purging zones (fetus s2212). Panels (A), (C), and (D) show a left‐lateral, a ventral, and a dorsal view, respectively, of the vagina of a ~13‐week‐old fetus. The vaginal plates are coded light‐green, the purging zone dark‐green, and the lumen of the vaginal part of the uterovaginal canal gray. Panel (C1) shows an identical view as panel (C), except that the bilateral purging zones and the transformational epithelium are left out to show the cranially wide and caudally narrow lumen of the vagina. Panel (B) shows a mid‐sagittal section through the vagina and cervix. The cervix region is lined by Müllerian‐duct epithelium, whereas the vagina is lined over its entire length by transformational epithelium. Panel (E) shows an oblique sagittal section, with on the left the maturing vaginal plate (panel (F); for a mature vaginal plate, see Figure 9E), the purging zone in the middle (panel (G)), and the zone with transformational epithelium in the wide cranial part of the vagina (panel (H)). The position of section “E” is shown as a dashed line in panel (D). Note that the maturing vaginal plate is still relatively thick and not yet made up just two facing epithelial layers. The position of the purging complex between the vaginal plates and transformational epithelium suggests that the purging front moves medially. Further note the trains of small sinusal cells (small arrows in panels (H)) at the base of the transformational epithelium. B: bladder; Cau: caudal; Cr: cranial; D: dorsal; L: left; MD: Müllerian duct; PZ: purging zone; R: right; SE: sinusal epithelium; TE: transformational epithelium; U: urethra; V: ventral; Vest: vaginal vestibule. Bars: 1 mm for reconstructions and 0.1 mm for sections.

Four female embryos with a crown‐rump length of 30–40 mm were available for study (Table 1). In none of these embryos did the Müllerian ducts establish an open communication with the urogenital sinus. The contact surface between both epithelia is limited by a relatively well‐defined intervening wedge of dense mesenchymal cells, which is largest on the right side (Figures 1A and 2B). The asymmetric positions of the mesenchymal wedge and the Müllerian head mirror a ~60° clockwise twist (seen from cranially) of the non‐fused caudal parts of the Müllerian ducts, so that it is mainly the left Müllerian duct that acquires a direct contact with the epithelium of the urogenital sinus (Figures 1B1–4 and S1). Meanwhile, the overlying urogenital‐sinus epithelium has formed a short urogenital epithelial plug in embryo s88 (Carnegie stage 23) and a somewhat longer funnel in the slightly more advanced fetus EYO295. This funnel physically contacts the Müllerian head (Figure 2C). The vanguard of these sinusal cells, so called because they originate in and stem from the urogenital‐sinus epithelium, breaks down the basement membrane of the Müllerian head, possibly in cooperation with the adjacent dense mesenchymal cells, and establishes a bridgehead (Figure 2D1–4). Although the Müllerian epithelium in the head of the uterovaginal canal loses its dorsal and ventral basement membranes and, to a lesser extent, its epithelial arrangement, its basement membrane remains intact laterally between the Müllerian head and the wide Wolffian ducts (Figures 3D–F, 4F–H, and 5B). Despite these cytoarchitectural changes in the Müllerian epithelium, the shape of the Müllerian duct and its epithelium initially hardly change, having an external diameter of ~400 × 125 μm and a wall thickness of ~35 μm (Figure 5). In summary, a zone of cell mixing invades the head of the uterovaginal canal but, apart from a clockwise helical growth in the caudal, non‐fused parts of the Müllerian ducts, the overall configuration of the tissues and their cell arrangement in the uterovaginal canal cranial to its head do not change appreciably during the 9th and 10th weeks of development. Remodeling of these parts of the uterovaginal canal will take place in the next 2 weeks (Figures 3, 4, 5). The central lumen inside the enlarged caudal end of the uterovaginal canal remains, nonetheless, a convenient landmark for the location of the Müllerian epithelium until the 13th week of development (Figures 2, 3, 4, 5, 6, 7).

### 3.3 The Fate of the Wolffian Ducts in Female Fetuses

**FIGURE 8:**
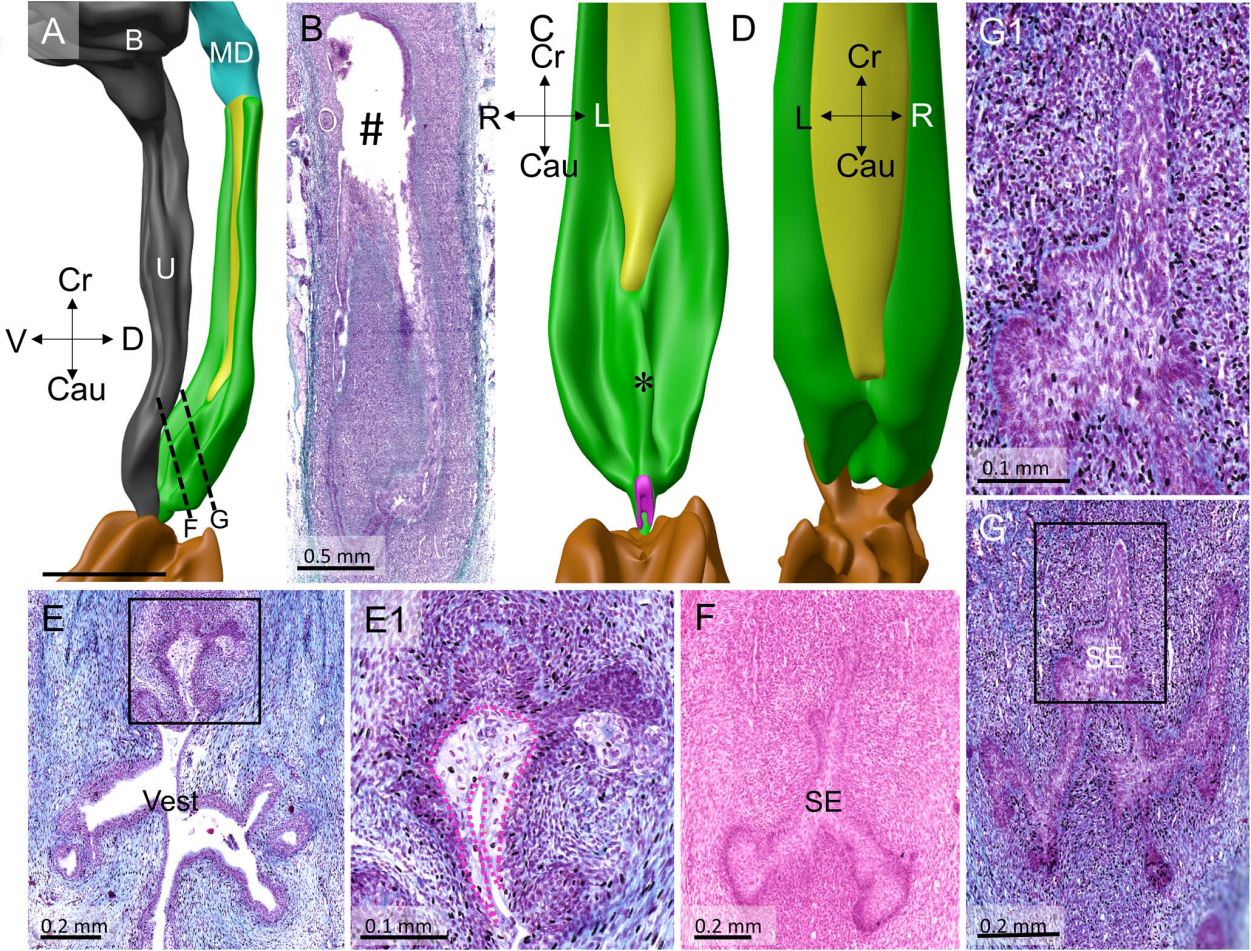
The caudocranial gradient in the development of the vaginal plates (fetus ME29). Panels (A), (C), and (D) show a left‐sided, a ventral, and a dorsal view of a reconstruction of a 14‐week‐old fetus. Green identifies the two lateral and the shorter medial vaginal plates (asterisk in panel (C)). The sinusal funnel, which can be identified by its clear cells, is visible caudally (panels (E) and (E1)). The caudal third of the vagina consists of vaginal plates that have developed subplates and are bordered by densely staining cells expressing a well‐developed basement membrane and periodical intumescences of randomly arranged cells (panels (F): near the funnel, (G) and (G1): in the middle of the dorsal plate). The vaginal lumen (#) is wide in the cranial half of the vagina (panel (B)). B: bladder; Cau: caudal; CC: clear cells; Cr: cranial; D: dorsal; L: left; MD: Müllerian duct; R: right; SE: sinusal epithelium; TE: transformational epithelium; U: urethra; V: ventral. Bars: 0.5 mm in the reconstructions and panel B; 0.2 mm in the other sections, except the detail (0.1 mm).

After the insertion of the Wolffian ducts into the ventral cloaca at Carnegie stage 13 (~4.5 weeks of development; Kruepunga et al. 2018), they retain their open connection with the urogenital sinus in female fetuses until week 12 (see below). The pseudostratified Wolffian epithelium has a smooth surface and a well‐developed basement membrane (Figures 1, 3, and 6). In the 9th week, the urogenital epithelium between and around both Wolffian outlets into the urogenital sinus differentiates into the so‐called “Wolffian‐crest” (“Wolffsche Kamm”; Kempermann 1931), or “clear” cells (Bulmer 1957), so named because the cytosol of these columnar cells stains markedly less intensely than that of the smaller basal cells of the urogenital epithelium (Figures 1, 3, 4, 5, 6, and 8). Around and between both Wolffian outlets, the urogenital epithelium consists of clear cells only and rests directly on the underlying wedge of dense mesenchyme. As a result, sinusal clear cells come to line the Wolffian outlets into the urogenital sinus (Figures 1, 3, and 4). Further away from the Wolffian outlets, the epithelium of the lateral wall of the urogenital sinus is becoming bilayered, with small, densely staining cells at the epithelial base and clear cells at the surface (Figures 3, 4, 5, 6). During the 10th week of development, the shape of a cross section of the urogenital sinus near the Müllerian tubercle changes from a crescent (Figures 1 and 2) into that of a butterfly (Figures 3 and 4), with the large dorsal “wings” flanking the Müllerian tubercle. The clear cells on top of the dense sinusal cells expand around the circumference of the dorsal wings of the urogenital sinus up to the two similar, but smaller protrusions in the ventral wall of the urogenital sinus (the ventral “wings”; Figures 3 and 4). Meanwhile, the Wolffian epithelium of the caudal ends of the Wolffian ducts regresses rapidly in female embryos. The more cranial parts of the Wolffian ducts have already disappeared in some (Figure 4), but not in all fetuses (Figures 6 and 7). More caudally, casts of dead cells are present in the part of the Wolffian‐duct lumen that is now lined by clear cells (Figure 4G,H). Cell degeneration in the distal Wolffian ducts at this stage was observed in earlier studies, but ascribed to their embedding in the (much wider) genital cord (Koff 1933, p. 74). Whereas Koff concluded that the dead cells block the exits of the Wolffian ducts into the urogenital sinus, we observed that they continue to exit into the urogenital sinus near the tip of the sinusal funnel (Figure 6A–E). This location deep inside the funnel originally belonged to the domain of the Müllerian tubercle (see next paragraph).

### 3.4 Invasion of the Müllerian‐Duct Epithelium by Urogenital‐Sinus Epithelium

The persistence of an intact basement membrane between Müllerian and Wolffian epithelium (first paragraph of the Results section) and the death of Wolffian epithelial cells that are in direct contact with the urogenital clear cells (previous paragraph) argues against a contribution of Wolffian epithelial cells to the definitive vaginal epithelium, as claimed by several investigators (e.g., Tourneux and Legay 1884; Mijsberg 1924; Kempermann 1931). On the other hand, the topographical relations that evolve in the 9th and 10th weeks of development indicate that sinusal and mesenchymal cells can break up basement membranes and subsequently infiltrate the Müllerian epithelium (Figures 2, 3, 4, 5, 6).

The intrusion of the urogenital‐sinus cells into the uterovaginal canal brings about an extensive remodeling of this region during weeks 10–12 of development (Figures 3, 4, 5, 6). In this period the expanding mesenchymal tissues of the genital cord engulf the Müllerian tubercle and transform it from a shallow prominence to the bottom of a funnel. The funnel is lined internally by basal urogenital‐sinus cells and is largely filled with clear cells (Figures 5, 6, 8, S2, and S3). During the remodeling, the epithelial perimeter of the urogenital sinus, including its folds, does not change appreciably (*cf*. micrometer bars in Figures 4 and 6 [~2 weeks apart in development]), but the dorsolateral and ventral folds of the urogenital sinus, which thus far bestow the shape a butterfly on transverse sections (Figures 3 and 4), have disappeared (Figure 6). Concurrently with the flattening of these folds, the diameter of the lumen of the urogenital sinus near the sinusal funnel (formerly the Müllerian tubercle) increases (Figure S2).

The basal epithelial cells in the funnel are directly continuous, via a narrow conduit, with phenotypically similar cells in the genital cord (Figure 5B). These cells are present as a double layer of small (cell diameter ~20 μm), intensely staining cells with relatively large nuclei that rest on an, at least initially, clearly visible basement membrane (arrows in Figure 5B). These cells differ from those in the funnel only in that they do not form a covering layer of clear cells. Instead, their apical sides engage in the formation of an epithelial double layer, with the cells facing each other in a tête‐à‐tête fashion. The double layer of sinusal cells separates and expands into an elliptical mass of cells where it contacts the head of the uterovaginal canal (Figure 5B,B1). The short and long axes of the mass measure ~110 and ~160 μm, respectively. Its outer cell layer surrounds a central group of randomly oriented cells.

In contrast to the sinusal epithelium, the epithelium of the head of the uterovaginal canal is fairly thick (~35 μm). Before the remodeling, this epithelium is characterized by a pseudostratified arrangement of its nuclei, a smooth luminal surface, and a well‐developed basement membrane (Figures 2B and 4D). This cellular arrangement is largely lost when the invading sinusal epithelial cells arrive and contact the epithelial cells of the head of the uterovaginal canal: the tall Müllerian epithelial cells become polymorphic and irregularly distributed, pycnotic cells appear, the basal membrane is interrupted at many locations, and regressively looking cells appear in its lumen. Nevertheless, the epithelial outline persists, largely due to the continuing presence of the central lumen of the uterovaginal canal apically and fragments of its basal membrane basally (Figures 3D,E, 4D–H, and 5B). No basement membrane is detectable at the boundary between the mass that is predominantly occupied by sinusal cells and the Müllerian cells of the uterovaginal head (white arrowheads in Figure 5B1). The location and phenotype of the cells in the caudal Müllerian duct relate these cells to those that we described in 8–10‐week‐old fetuses as resulting from the mixing of the Müllerian, sinus, and dense‐mesenchymal cells (*cf*. Figure 5B1 with Figures 2C and 4E–H).

The epithelial conversion from the Müllerian to this “transformational” phenotype in the adjacent, more cranial ~30% of the uterovaginal canal is typically accompanied by the acquisition of a much wider, but shallow lumen (Figures 6, 7, 8). Its epithelium is also thinner (~20 μm), but remains made up of the same disparate, randomly arranged cell mix that is found in the head of the canal. Furthermore, the basement membrane is interrupted at many locations, and small protruding cells at the luminal surface seem to be in the process of being extruded. In addition to the random arrangement of cells, the markedly thinner epithelium in the more cranial parts of the vagina compared to its caudal head is striking. This difference exists as early as the formation of the uterovaginal canal (Figures 1, 3, 6, and 7). The main difference between the 10th and 12th weeks of development is, therefore, the rapid caudal‐to‐cranial expansion of the transformational epithelium in the cranial part of the uterovaginal canal.

The part of the uterovaginal canal in which cell mixing is microscopically observed is restricted to its head (~20% of the length of the uterovaginal canal) in advanced 10‐week‐old fetuses (Figure 4), but has extended cranially to a level that corresponds to ~40% of the length of the uterovaginal canal at 11 weeks (Figure 5), and to ~50% at 12 weeks (Figure 6). Fifty percent of the uterovaginal canal is equivalent to the length of the vagina at this stage. This description demonstrates that the intrusion of the uterovaginal canal that is first observed in the 9th week of development (Figure 2) continues and progresses in a cranial direction at a rate of ~15% of the total length of the uterovaginal canal per week. The conversion of Müllerian epithelium to a transformational epithelium has extended across the caudal ~50% of the uterovaginal canal, or the entire vagina, in 12‐week‐old fetuses (Figure 6).

### 3.5 The Formation of the Vaginal Plates

In the 12th week, the sinusal funnel remains present in a nearly unmodified form. The small sinusal cells that line the funnel rest on a well‐developed basement membrane, while more superficial clear cells largely fill the lumen. At the funnel tip, sinusal cells are producing a medial and two lateral double‐layered plates, in which the epithelial cells are aligned tête‐à‐tête (Figure 6). Only a few regressive‐looking cells (dense nucleus and poorly staining cytoplasm) are present between both lateral epithelial plates. Koff labeled these double‐layered plates of epithelial cells the “vaginal plates” (Koff 1933) but applied this name to the lateral cell plates only and did not separately describe the medial cell plate. The lateral vaginal plates originate just caudally to the outlets of the Wolffian ducts as lateral evaginations of the sinusal funnel (Figure S2). The vaginal plates expand cranially by extending and widening the already formed caudal part of the vaginal plates on the lateral side of the uterovaginal canal and, at some distance, on the medial side of the Wolffian ducts. All three vaginal plates are supported by a well‐developed basement membrane.

As in the previous stage, the head of the uterovaginal canal of the 12‐week‐old fetus ends near the narrow tip of the funnel (Figure 6G). A small, centrally localized mass of randomly oriented cells that have lost their basement membrane entirely is present in between the sinusal funnel and the remaining part of the head of the uterovaginal canal (Figure 6G). This median agglomerate of cells occupies the same location as the median mass of sinusal cells near the head of the uterovaginal canal that we described in the previous stage (Figure 5). Since we find only one intumescence at 11 and 12 weeks, which is in close contact with the uterovaginal epithelium (Figures 5B and 6G), this intumescence could well represent the slow dissolution of the dense mesenchymal wedge seen between 8 and 10 weeks of development (Figures 1B, 3D–F, and 5F–H). Unfortunately, no lineage data in, for example, mice are available at present, so that we cannot weigh this hypothesis. Of note, the sinusal cells of the funnel and the epithelial cells of the remaining part of the head of the uterovaginal are not separated by a basement membrane (Figure 6F,G).

**FIGURE 9:**
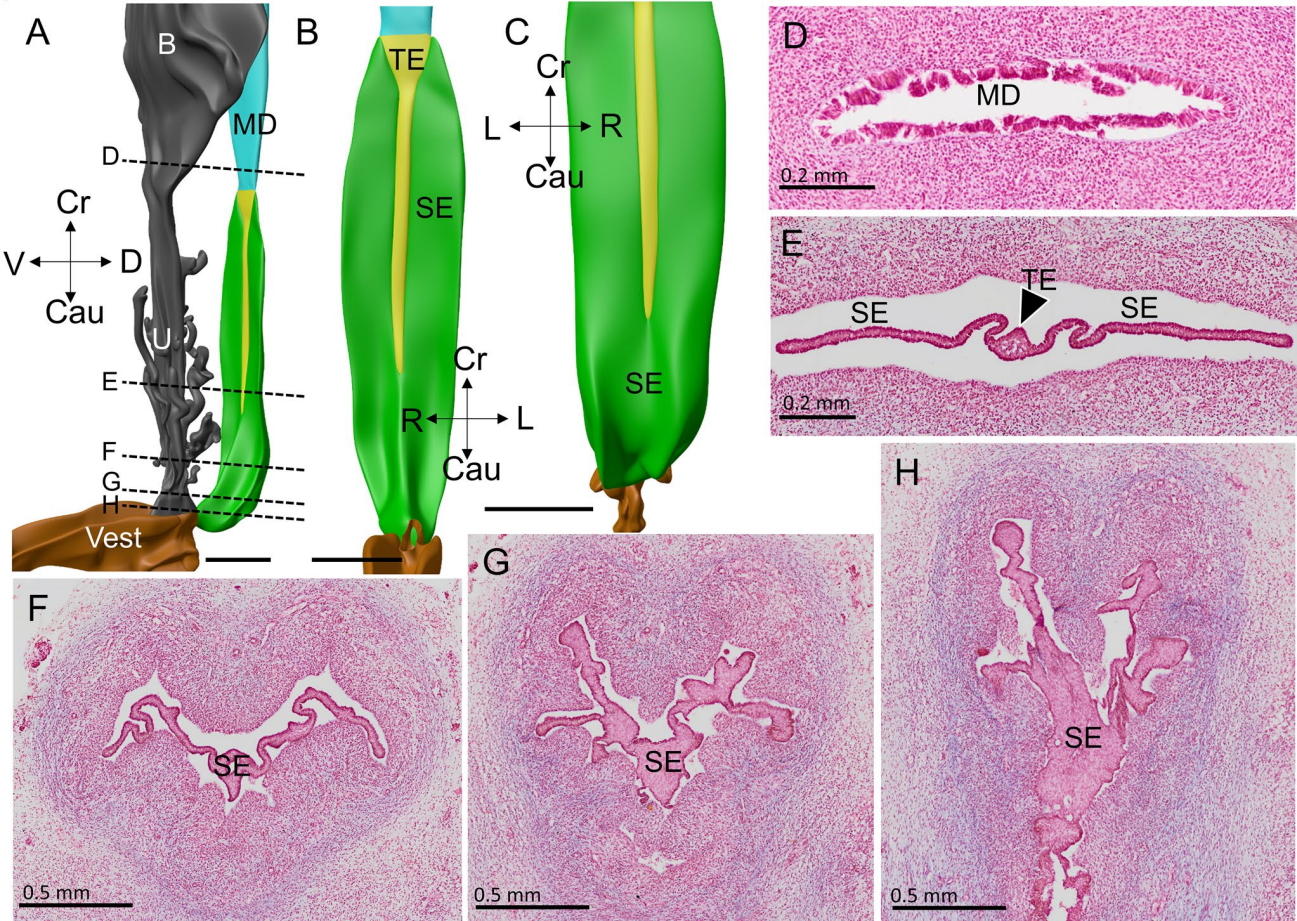
The mature vaginal plates occlude the vaginal lumen (fetus s2392). Panels (A–C) show a left‐lateral, a ventral, and a dorsal view of the vaginal epithelium at ~15 weeks. The specimen is somewhat macerated, so that the epithelium has detached from the wall of the vagina (see main text for a discussion of this artifact). Panel (D) shows the relatively narrow lumen of the uterovaginal junction. Panel (E) shows a more caudal section of the vagina. The non‐epithelial cells in the midline are not integrated in the vaginal plate and indicate that the cell replacement is not yet final (see yellow median band in panels (A–C)). This area increases in size going cranially. Panel (F–H) shows the vaginal plate in the caudal third of the vagina: There is a gradient in the degree of branching of the vaginal plates and this gradient declines going cranially. B: bladder; Cau: caudal; Cr: cranial; D: dorsal; L: left; MD: Müllerian duct; R: right; SE: sinusal epithelium; TE: transformational epithelium; U: urethra; V: ventral. Bars: 1 mm for the reconstructions, 0.5 mm for sections (F–H), and 0.2 mm for sections (D) and (E).

Just beyond the tip of the sinusal funnel, the lumen of the uterovaginal canal is still relatively narrow (diameter: ~150 μm), the lining epithelium is relatively thick (~35 μm), and the lumen contains regressive cells (Figure 6H), which are characteristics of its head. Further cranially, its lumen widens (Figures 5C and 6C1) but, like the caudal part of the uterovaginal canal, its epithelial cell arrangement is irregular, contains pycnotic nuclei, and harbors cell fragments in its lumen (Figures 6J and 7H). This cranial portion is, like that in the 11‐week‐old fetus, further characterized by a relatively thin epithelial layer (~20 μm) and a relatively large oval lumen (Figure 6J). Its short and long axes measure ~2 and ~5 mm, respectively. The thick epithelium of the head occupies the caudal ~20% of the entire uterovaginal canal, whereas the more cranially thin “transformational” epithelium that surrounds the enlarged Müllerian lumen (Figure 6C) accounts for ~30%. The transformational epithelium of the uterovaginal canal is continuous laterally with the lateral vaginal plates of double‐layered epithelium (Figure 7H), whereas the medial vaginal plate has ended at ~20% of the uterovaginal canal. The lateral vaginal plates with their double layer of tête‐à‐tête aligned epithelial sheets are widest caudally and become gradually narrower going cranially (Figure 6B–D). These vaginal plates end where uterovaginal epithelium continues to express the typical Müllerian phenotype homogeneously. Topographically, the changeover of the transformational, future vaginal epithelium to the classical Müllerian epithelium is found at the level of the bladder neck (Figures 7, 8, 9).

### 3.6 The Purging of the Transformational Epithelium

During the 13th week, differentiation of the vaginal epithelium continues to proceed from caudal to cranial, as described for weeks 11 and 12. The cellular arrangement of sinusal cells in the funnel remains unchanged and still supports the sheet of transformational epithelium that surrounds the lumen of the uterovaginal canal (Figure S3B). The caudal and lateral parts of this sheet are made up of the small, densely staining cells of the double‐layered vaginal plates described by Koff (Figure 7C,D). The vaginal plates are widest caudally, where they border the head of the uterovaginal canal laterally. Here, the vaginal plates have developed a few intumescences, which are aggregates of small dark‐staining cells at their periphery and bigger, less intensely staining cells in their center. These cells resemble the ones found in the single intumescence we described in the 11‐ and 12‐week‐old fetuses. The peripheral cells of the intumescences are not supported by a basement membrane, whereas the double‐layered sheets of the vaginal plates are (Figure 7E,F). Lateral to the head, the sheets of the vaginal plates also produce secondary protrusions that increase their surface and volume.

The transformational epithelium of the wide oval lumen of the uterovaginal canal in the cranial part of the vagina has matured into a continuous basal layer of small sinusal cells (“train”; Figure 7H,H1). This basal layer is covered on its apical side by randomly oriented cells, many of which contain densely stained nuclei or are pycnotic. Some cells protrude into the lumen. More laterally, the transformational epithelium thickens, its surface becomes more rugged, and the vaginal lumen becomes shallower (Figure 7G). The zone is also lined by a basal layer of small cells. The nuclei in the top layer are hyperchromatic, oblong, and mostly oriented parallel to the luminal surface. Laterally, this new zone ends rather abruptly where the dorsal and ventral layers of rugged epithelium surrounding the vaginal lumen merge and continue as the double‐layered lateral vaginal plates. Within 10–15 cell diameters, the thickness of the merged epithelia declines two to threefold (Figure 7E). Visually, the epithelium of the short junctional area resembles a honeycomb. Many large, but regressive‐looking cells with central pycnotic nuclei and a non‐staining rim of cytoplasm have been apparently eliminated because their number drops precipitously in the adjacent vaginal plate. These features constitute a relatively easily identifiable landmark for the boundary. We have named this zone in the wall of the developing vagina the “purging zone,” because the adjacent vaginal plate contains far fewer regressive cells and a much more homogeneous cell composition.

A “pure” two‐layered tête‐à‐tête arrangement, as present in the vaginal plates of 12‐week‐old fetuses (Figure 6) is, nonetheless, not yet present in the part of the vaginal plate that borders the purging zone. This observation suggests that this part of the vaginal plate still shows some immature features. When we compare the vaginal plate of the 13‐week‐old fetus at caudal and more cranial positions (Figure S3), the more mature caudal vaginal plate is typically bi‐layered, whereas the more cranially located vaginal plate is wider and still contains relatively small cells with pycnotic nuclei and nonstaining cytoplasm in its center. The transition from a multi‐layered to a two‐layered vaginal‐plate configuration is visible in a single caudal section near the midline (Figure S3). This oblique section shows the sinusal funnel, supporting the mature two‐layered vaginal plate. More cranially, the vaginal plate first shows immature properties (wider, with centrally degenerating cells) and then assumes all the characteristics described above for the purging zone. Figure S3A also shows the cranially wide uterovaginal canal with the epithelial characteristics of transformational epithelium. In summary, our findings in the 13‐week‐old fetus indicate that the vaginal plates have become wider and that their boundary has moved medially. We posit that the purging zone identifies the location where the non‐sinusal epithelial cells are eliminated from the vaginal epithelium.

The 14‐week specimen that we studied exhibits a very well‐developed vaginal‐plate system. The caudal‐most part of the vagina still reveals its origin by the presence of clear cells in its lumen (Figure 8E,E1). Cranial to the part containing these clear cells, the layered non‐luminal vaginal plates consist, as in the previous 2 weeks, of a medial and two lateral, well‐developed epithelial folds (Figure 8F). The relatively short medial vaginal plate corresponds in length and position with the head of the uterovaginal canal. This non‐luminal caudal part of the vaginal epithelium accounts for ~30% of vaginal length in the 14‐week fetus. In the caudal part of the developing vagina, the vaginal plates produce at seemingly random locations short secondary plates with locally small dilations. These secondary plates profoundly increase the surface area of the caudal vagina. The outer cell layer of the dilations consists of darkly staining cells, whereas the more centrally located cells are larger and have lighter‐staining nuclei and cytoplasm but are interspersed with pycnotic nuclei (Figure 8G,G1). A well‐developed basement membrane supports the predominantly double‐layered vaginal plates, but the basement membrane between the dilations and the surrounding connective tissue of the genital cord contains many interruptions. The connective tissue itself contains healthy‐looking cells but also numerous cells with pycnotic nuclei. The dilations, therefore, appear to represent a three‐dimensionally more branched purging zone than the linear caudo‐cranially oriented purging zone that we described in the previous stage. In contrast to its caudal portion, the more cranial portion of the vagina still has a real lumen (Figure 8B). Unfortunately, the coronal orientation of the sections at this location only yields tangential sections of the epithelium. The junction with the developing cervix is relatively narrow and is covered with typical Müllerian cells (not shown).

In the 15th week, finally, the transformation of the epithelium of the vaginal wall is virtually complete and the vaginal lumen is occluded (Figure 9). The vaginal wall is ~twofold thinner than that of the cervix and uterus (Figure S4). The vaginal epithelium itself is arranged as a nearly complete tête‐à‐tête bilayer of sinusal cells (Figure 9B,C). In the midline, the bilayer is interrupted at places (Figure S4B1), probably because the purging process still has to be completed there. We, therefore, assume that the epithelium of the entire wall of the vagina is of urogenital origin after 16 weeks of development. More cranially in the forming vagina, the epithelium has also transformed to a tête‐à‐tête arranged bilayer of sinusal cells. Its junction with the cervix is characterized by a substantial increase in wall thickness and a decrease in the diameter of the uterovaginal canal (Figure S4). Whereas the three vaginal plates still meet at perpendicular angles in 12‐week‐old fetuses (Figure 7E), their profile has changed into that of the letter “W” in the 14‐ and 15‐week‐old specimens (Figures 8G and 9F). This change in configuration implies that the cell masses in between these cell plates have expanded in a caudoventral direction. The cervical epithelium is typically Müllerian (Figure S4).

### 3.7 Epithelial Changes Between 16 and 20 Weeks of Development

**FIGURE 10:**
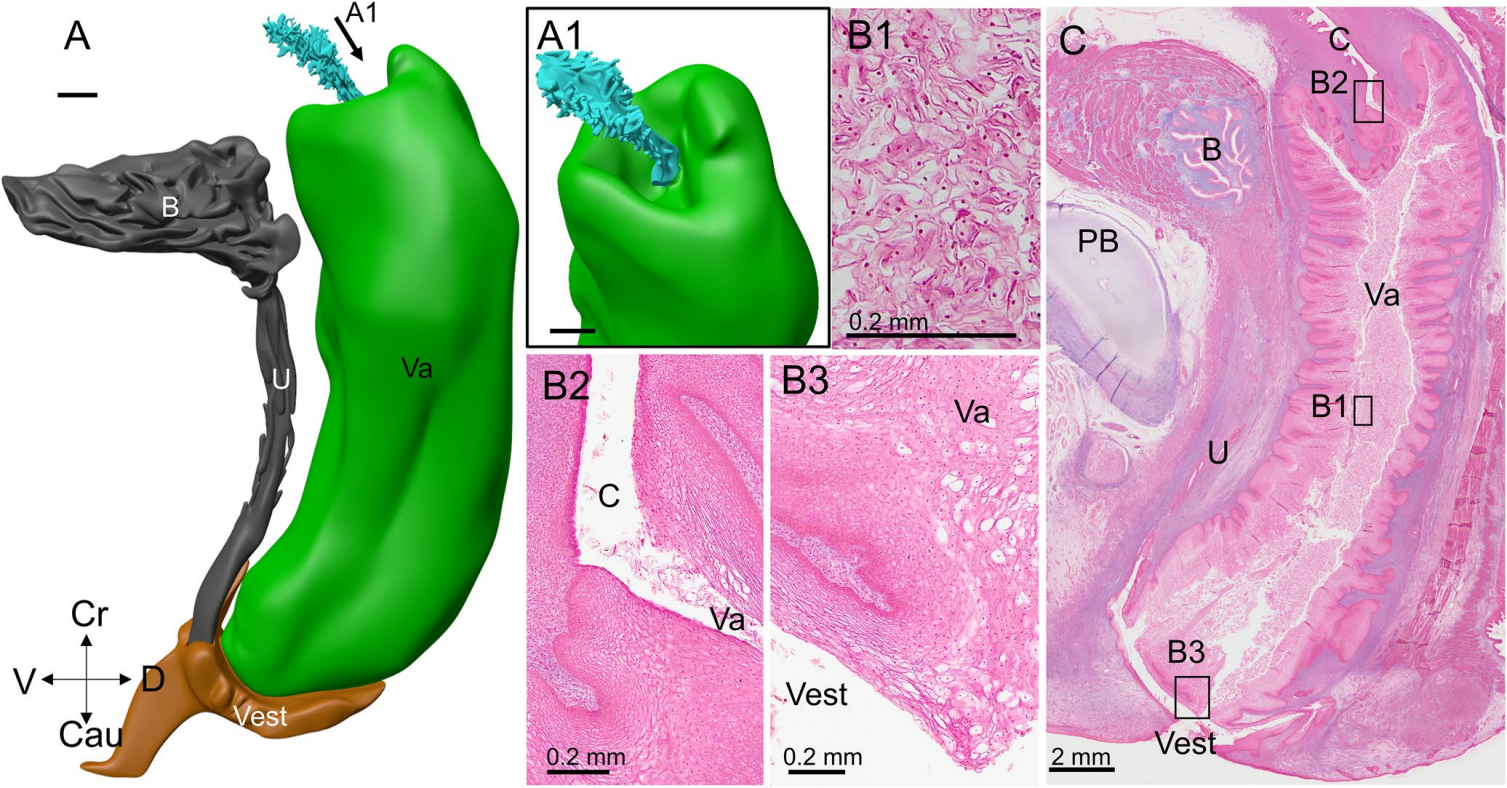
The differentiation of the fetal vagina lined by urogenital epithelium (fetus s2290). Panels (A) and (A1) show a left‐lateral and left‐superior view of the vagina (green), cervix (cyan) and vestibule (brown) at ~20 weeks. A midsagittal section of the vagina is shown in panel (C). It shows the locations of the magnifications (B1–3) (squares). The “lumen” of the vagina is filled with mostly dead cells (panel (B1)). Panel (B2) shows the sharp epithelial transition between the vagina and cervix, while panel (B3) shows the vaginal vestibule. B: bladder; C: cervix; Cau: caudal; Cr: cranial; D: dorsal; PB: pubic bone; U: urethra; V: ventral; Va: vagina; Vest: vaginal vestibule. Bars: 2 mm in reconstructions and section C, and 0.2 mm for sections B1‐3.

Unfortunately, we did not have access to specimens in the age groups between 16 and 20 weeks of development. In the 20th week, the vaginal and cervical epithelium have differentiated (Figure 10B2). The vagina has become a large organ lined by a stratified squamous epithelium with a hypertrophic appearance. It is made up of a basal layer of 1–2 small, densely staining cell layers that are mostly oriented perpendicularly to their attachment surface. Towards the center of the vagina, these cells gradually transform into poorly staining cells with increasingly pycnotic nuclei (Figure 10B1). The center of the vagina is filled with mostly dead cells.

The cervical canal is tiny in circumference relative to the lumen of the vagina and lined with simple columnar epithelium (Figure 10A). The 3–4 mm of this epithelium that is closest to the vagina rests on a layer of 2–3 mesenchymal cells with a light‐staining cytoplasm and relatively large nuclei. These cells, in turn, rest on densely packed mesenchymal cells, like the more cranial epithelium of the cervix. The squamocolumnar border of the vagina and the cervix is sharp (Figure 10B2). At the junction of the two epithelia, the mesenchymal cells with weakly stained cytoplasm also extend underneath the vaginal epithelium.

The developmental anatomy of the vaginal vestibule was described elsewhere (Hülsman, Gao, et al. 2025b). At this stage of development, its lumen is a largely virtual space with apposed lateral walls. The boundary between keratinizing and non‐keratinizing epithelium is clearly visible by a change in color (Hülsman, Gao, et al. 2025b). At 15 weeks of development, radial folds mark the entrance to the lumen and the caudal part of the vagina itself (Figure 9A,F). This configuration resembles that in the adult. Because of tissue damage, it is not entirely clear whether the entrance to the lumen of the vagina at 20 weeks is open or closed by a layer of dead epithelial cells (Figure 10B3).

### 3.8 The Position of the Müllerian Tubercle

**FIGURE 11:**
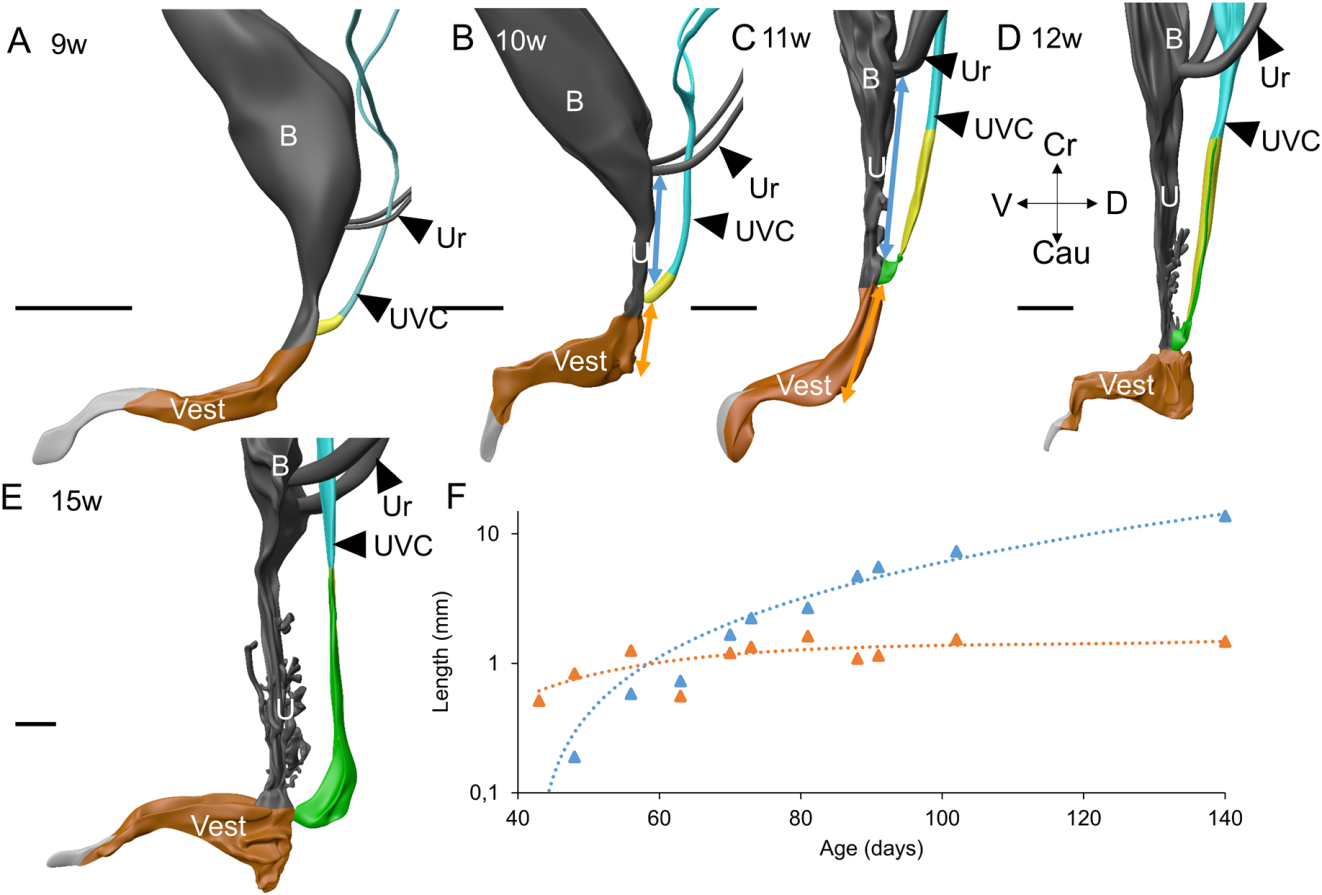
Differential growth underlies the perineal position of the definitive vagina. Panels (A–E) show a left lateral view of the urogenital sinus, urethra, bladder, and Müllerian duct between 9 and 15 weeks of development (S89, S4908, S1743, S2383, and S2392). The distance between the ureteric orifice and the Müllerian tubercle (blue double‐headed arrow in panels (B) and (C)) increases exponentially during this period (blue symbols, panel (F); note logarithmic *Y*‐axis). The graph reveals two growth phases, with the break at 9–11 weeks of development. The break appears to coincide with the onset of the purification of the vaginal epithelium from regressive Müllerian cells. The distance between the Müllerian tubercle eminence and the urogenital “heel” (orang double‐headed arrow in panels (B) and (C)) on the other hand hardly increases (orange symbols, panel (F)). B: bladder; Cau: caudal; Cr: cranial; D: dorsal; Ur: ureters; U: urethra; UVC: uterovaginal canal; V: ventral; Vest: vaginal vestibule. Bars: 1 mm.

We have described the penetration of the urogenital‐sinus cells into the epithelium of the uterovaginal canal and the subsequent removal of the Müllerian epithelial cells without reference to the topography of the vagina in the pelvis. The longitudinal growth of the uterovaginal canal and the urethra, the corresponding part of the urogenital sinus, is responsible for the downward relocation of the Müllerian tubercle to its definitive position outside the lesser pelvis. The Müllerian tubercle reaches the pelvic floor in fetuses of 10–11 weeks of development (oblique blue lines in figure 14 of Hülsman, Köhler, et al. 2025a) and its near‐definitive position below the pelvic floor at 15–20 weeks. Figure 11 shows that the downward growth rate of the Müllerian tubercle is biphasic, with the ~twofold decline coinciding with the appearance of the vaginal plates in the 12th week. The second phase of growth appears to end when the replacement of the Müllerian epithelium of the vagina is completely replaced by urogenital epithelium shortly after 15 weeks. The distance between the Müllerian tubercle and the dorsal bottom of the vaginal vestibule, on the other hand, increases steadily but much slower.

### 3.9 The Vaginal Fascia

Up to 9 weeks of development, only the head of the uterovaginal canal is embedded in a denser mesenchyme than the remaining, more cranial part. Between 9 and 12 weeks, the entire uterovaginal canal becomes embedded in a more peripheral mesenchymal sheath, the “genital cord.” The dense connective‐tissue boundary of the genital cord is located outside the dense mesenchymal condensation that brings about the asymmetry in the head of the uterovaginal canal. The border of the uterovaginal canal becomes less pronounced further cranially along the uterovaginal canal. At 13 weeks, the mesenchyme that surrounds the uterovaginal canal is still easily identifiable. However, the fascial layer around the head of the uterovaginal canal separates from that also surrounding the urogenital sinus or, later, the urethra, as the vagina extends caudally during its “descent” (figure 14 in Hülsman, Gao, et al. 2025b). The course of the, in fetuses still well‐developed lower part of the urethral sphincter, the urethrovaginal sphincter, reveals that most caudally, the adventitias of the urethra and vagina are still closely adjacent structures (Figures 7, 8, 9).

## 4 Discussion

Our findings show that the development of the female genital tract can be divided into an early phase in which its craniocaudal growth predominates, and a later phase which is characterized by its mostly caudocranial direction of differentiation. The early phase is described in the accompanying report (Hülsman, Köhler, et al. 2025a). Unexpectedly, our observations on late Müllerian duct development and differentiation, particularly the replacement of Müllerian by sinusal epithelium, differ greatly from available accounts.

### 4.1 Epithelial Differentiation of the Uterovaginal Canal Proceeds in Caudocranial Direction

In contrast with the growth of the Müllerian duct from its site of specification in the upper thoracic region to the dorsal wall of the urogenital sinus (Hülsman, Köhler, et al. 2025a), epithelial differentiation of the uterovaginal canal proceeds from the Müllerian tubercle in cranial direction. At the beginning of the 9th week, the sinusal epithelium on and around the Müllerian tubercle starts to differentiate into a layer of small, intensely staining basal cells and a more superficial layer of columnar epithelial cells with a poorly‐staining cytoplasm (“äussere and innere Epithellamelle”; Kempermann 1931). The “inner” cells are better known as “clear‐staining” (Bulmer 1957) or “clear” cells (Davies and Kusama 1962). Although the term “clear cells” when used in a pathological context, including that of the female genital tract, usually refer to an adenocarcinomas (Matias‐Guiu et al. 1997), we use the term to indicate the urogenital cells with these staining properties that line the dorsal and, to a lesser extent, the lateral walls of the urogenital sinus, but also expand over the dorsal wall of the developing urethra. Because of their prominent presence in the midline of 10–11‐week‐old fetuses, this midline is also known as the “Wolffschen Kamm” (Wolffian ridge) (Kempermann 1931). The clear cells remain associated with the junction of the Müllerian ducts and the urogenital sinus and, hence, their presence is an excellent marker of this site until at least 14 weeks of development (Figure 10E,E1; figure 5 in Davies and Kusama 1962).

A critical step in the differentiation of the vaginal epithelium is the not previously described penetration and spreading of the small basal urogenital epithelial cells into the Müllerian epithelium and the adjacent mesenchyme of the genital cord. In the 9th week, the head of the uterovaginal canal exhibits a helical growth in a clockwise direction when looking downward. This asymmetric growth is most pronounced in the voluminous head of the uterovaginal canal and ends when the sinusal cells start to invade the Müllerian epithelium. The helical growth confers a ventral position on the left‐sided Müllerian duct, which allows it to directly contact the wall of the urogenital sinus, whereas a mesenchymal wedge blocks the contact of the right‐sided duct with the sinus epithelium. Helical growth of a tubular structure in a clockwise direction, when looking distally along its long axis, is also observed during the looping phase of the heart (Hikspoors et al. 2022) and that of the small intestine (Soffers et al. 2015). While helical growth may be necessary to accommodate looping of tubular structures in a confined space (Le Garrec et al. 2017), the mechanism for its requirement to bring about proper contact between the sinusal and Müllerian epithelia is presently unclear. In this respect, it is of interest that the vagina of dolphins has a clockwise spiraling lumen (Orbach et al. 2020) and that in women with uterus didelphys and obstructed hemivagina, the affected hemivagina was found in two‐thirds of cases on the right side (Vercellini et al. 2007).

Penetration and spreading of basal urogenital epithelial cells in the vaginal Müllerian epithelium reach the uterovaginal junction at ~11 weeks. The penetration of these “foreign” cells into the vaginal epithelium itself is generally accepted, but root and route of the penetrating cells have long been argued. Earlier studies, which proved right, had relied on the typical small‐cell phenotype of the penetrating cells (Vilas 1932; Meyer 1934, 1936, 1937). However, it is only the relatively recent immunohistochemical staining of the replacing epithelium as FOXA1‐positive/PAX2‐negative (Robboy et al. 2017) that has practically settled its origin as urogenital. We have tracked the penetration and spreading of the sinusal cells into the Müllerian epithelium in the 9th–12th week, and the subsequent elimination of the Müllerian cells from the mixed cell population by the vaginal plates in the 12th–15th week. We have named the mixed cell population a “transformational epithelium” since it is subject to a change in its cell population but retains the boundaries of the original Müllerian epithelium. After completion of this cell replacement in the 16th week, the vagina temporally resembles a deflated balloon that is lined by small epithelial cells that are adhering apically in a tête‐à‐tête alignment. Based on these observations we propose a novel 2‐phase model for vaginal epithelial differentiation, which we named the “invade and purge” model (next section). Other investigators have described the beginning and the final outcome of this process. Bulmer, for example, described the process as “An outer proliferation of darkly staining cells which grew dorsally, displaced the Müllerian cells from the Müllerian tubercle and extended cranially as an epithelial plate, subsequently canalized to form the vagina” (Bulmer 1957), while Koff's summary is even briefer: “The columnar epithelium lining the caudal portion of the uterovaginal canal becomes stratified.” Davies and Kusama observed a junction between Müllerian and “transformational” epithelium (their Figure 6) that mirrors our Figure 7B, but described it as “becoming characteristic of the stratified squamous epithelium in the vaginal region” (Davies and Kusama 1962). The conversion of the caudal half of the uterovaginal canal into the vaginal plates is completed at ~16 weeks (Koff 1933). To our knowledge, however, the first phase of vaginal epithelial differentiation has not yet been characterized thus far, while the dynamics of the second phase have hardly been discussed.

### 4.2 Replacement of the Vaginal Epithelium: The “biphasic vaginal epithelial invasion and purging model” (Figure 13)

Vaginal epithelial differentiation starts in the first part of the 9th week of development, when urogenital epithelial cells penetrate the head of the uterovaginal canal. Possibly together with the dense mesenchymal cells, these “sinusal” cells bring about a partial breakdown of the Müllerian basement membrane, so that sinusal, Müllerian, and mesenchymal cells can mix and interact. As a result, the strict arrangement of epithelial cells is lost and a large part of its basement membrane is absent, but the epithelial compartment as such remains recognizable. The penetration of the sinusal cells into the Müllerian epithelium of the vagina (the “transformation”) takes ~3.5 weeks to complete from caudal to cranial and reaches the uterovaginal junction in the 12th week of development. The head of the uterovaginal canal initially retains its epithelial thickness and narrow lumen, even though the invading cells change its cellular composition and arrangement. In the 11th week, the more cranial ~80% of the vaginal length acquires a wider, but shallow lumen that is lined by randomly arranged small cells that resemble those seen in the caudal portion of the vagina. Like more caudally, the transformational epithelium locally loses its basement membrane. The transformational epithelium in the cranial vagina differs from that in the caudal vagina mainly by the reduction of its thickness to ~60%. This epithelial thinning probably explains why remnants of the typical Müllerian epithelial cells, which are columnar in the caudal vaginal epithelium, are hard to find in the cranial part of the vagina.

In 12‐week‐old fetuses, the small sinusal cells of the funnel also penetrate the genital‐cord mesenchyme to form a mediodorsal and two lateral epithelial folds (Figure 6). These folds consist of almost pure sinusal cells. The mediodorsal fold is confined to the caudal ~25% of the vagina and corresponds topographically to the head of the uterovaginal canal. In contrast, the lateral folds flank the vagina along its entire length but are initially widest in its caudal part. Furthermore, the lateral folds become longer and, in particular, more caudally, wider and oriented in a dorsolateral direction. During the next week, these folds themselves develop smaller secondary folds in the caudal end of the vagina, while all folds display periodic dilations. The cells that form the primary and secondary folds are P63‐positive (figure 3 in Fritsch et al. 2012), and also FOXA1‐positive and PAX2‐negative (figure 21 in Robboy et al. 2017). These staining properties identify them as proliferating and differentiating sinusal cells, respectively. The crests of the medial and lateral folds of the caudal vagina form as bilayered sheets of tête‐à‐tête arranged deflated epithelium. Their expansion proceeds at the expense of the transformational epithelium. The lateral bilayered sheets are known as the “vaginal plates” (Koff 1933), while the medial sheet is only present in the caudal part of the vagina and was not given a name thus far.

The elimination of Müllerian and mesenchymal cells, which occurs just medial to the advancing vaginal plates, produces a thick epithelial ripple that contains many regressive and pycnotic cells. The selective removal of the apparent remnants of Müllerian and mesenchymal cells that made up the population mix of the transformational epithelium (see above) proceeds from caudal to cranial and from lateral to medial. The caudal‐to‐cranial progression of the replacement can be deduced from the cranial expansion of the wide part of the vaginal plate in fetuses of 12–15 weeks. Similarly, the lateral‐to‐medial progression of the replacement can be deduced from the increasing width with time of the vaginal plate at the expense of the zone of transformational epithelium, and from a few persisting midline remnants of PAX2‐positive Müllerian epithelial cells between the advancing vaginal plates at 15 weeks (Figures 11E and S4B1; figure 21 in Robboy et al. 2017). It is well conceivable that the medial vaginal sheet, which is morphologically very similar to the lateral vaginal plates, is responsible for the purging of Müllerian epithelial cells in the head of the uterovaginal canal. The removal of Müllerian cells from the vaginal epithelium and the associated production of a deflated vaginal lumen at ~16 weeks completes the second phase of vaginal epithelial remodeling. The deflated vaginal lumen with the associated tête‐à‐tête arrangement of the epithelial cells probably corresponds to the occlusion of the vaginal lumen that is usually described to arise towards the end of the first half of pregnancy (Vilas 1932; Meyer 1937; Matejka 1959; our specimen suffered from epithelial dehiscence, but figure 55a in Meyer 1937 and figure 5 in Davies and Kusama 1962 show similar sections of intact specimens). Apparently, this tête‐à‐tête adhesion is not very strong, because it permitted casting the vaginal lumen without apparent damage (Terruhn 1980).

### 4.3 The Completion of the Replacement of Müllerian by Sinusal Cells in the Vaginal Epithelium

**FIGURE 12:**
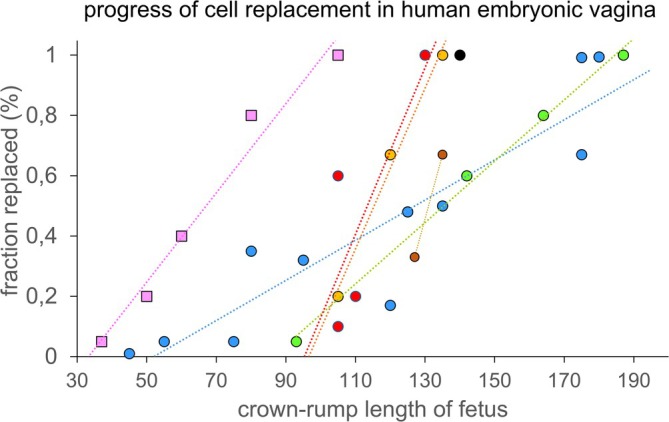
Timeline of the completion of the replacement of Müllerian by urogenital cells in the vaginal epithelium. The *X*‐axis represents the crown‐rump length of the fetuses, while the *Y*‐axis indicates the fraction of the vaginal epithelium with complete replacement. For a correlation between crown‐rump length and age of the fetus, see (Hülsman, Gao, et al. 2025a). The population of the vagina with transformational epithelium reaches the uterovaginal junction at ~12 weeks (square pink dots). The brown (Vilas 1932), orange (Kempermann 1935), blue (Meyer 1938), black (Bulmer 1957), green (Robboy et al. 2017), and red (present study ) circular dots show that the subsequent replacement of Müllerian by urogenital epithelium begins in the caudal‐most part of the vagina at 10–13 weeks (70–90 mm CRL), and spreads caudocranially to reach the uterovaginal junction at 15–19 weeks (130–180 mm CRL). The estimates of the duration of the replacement of the Müllerian by urogenital epithelium vary from 3 to 9 weeks, with a median of 5 weeks. For details, see main text.

The timeline of the replacement of the Müllerian by sinusal cells can be followed by identifying the respective cells morphologically (this study; Vilas 1932; Meyer 1934, 1936, 1937; Kempermann 1935; Bulmer 1957). The most extensive quantification was carried out by Meyer (p. 556 in Meyer 1938 and our Figure 12). The purging of Müllerian cells begins with the formation of vaginal plates in the caudal‐most part of the vagina at ~12 weeks (~85 mm CRL) and spreads caudocranially to reach the uterovaginal junction at ~16 weeks (~140 mm CRL (this study); Vilas 1932; Kempermann 1935; Bulmer 1957) or at 18–19 weeks (~170 mm CRL; Meyer 1938). More recently, Robboy et al. used antisera against the transcription factors PAX2 (expressed in Müllerian epithelium) and FOXA1 (expressed in sinusal epithelium) to follow the replacement of Müllerian by sinusal epithelium (Robboy et al. 2017). Their data position the completion of the replacement at 19–20 weeks. Our data suggest that the transformation proceeds more rapidly and is complete in fetuses of ~16 weeks. The quantitative discrepancies are probably accounted for by the variation in length (crown‐rump length) of the specimens: in Meyer's table, vaginal transformation in two 135 mm fetuses has proceeded to ~50% and ~95%, and in two 175 mm fetuses to ~65% and ~90%. Similarly, Robboy's CRL‐fetal age correlation deviates by ~2 weeks from our pooled average of six studies (Hülsman, Gao, et al. 2025a) between 20 and 34 weeks (Robboy et al. 2017). In view of the shallow inclination of his correlation graph between the crown‐rump length of a fetus and the fraction of replaced cells in the uterovaginal canal epithelium, Meyer's data seem to unintentionally combine the results of the penetration and purging phases of the sinusal transformational cells (Meyer 1938). On the other hand, the data of Vilas, Kempermann, and Bulmer indicate their specimens behaved much like ours with respect to the completion of the replacement. The accuracy of our reference line for the relation of crown‐rump lengths with weeks of development after fertilization is discussed in Hülsman, Gao, et al. (2025a).

**FIGURE 13:**
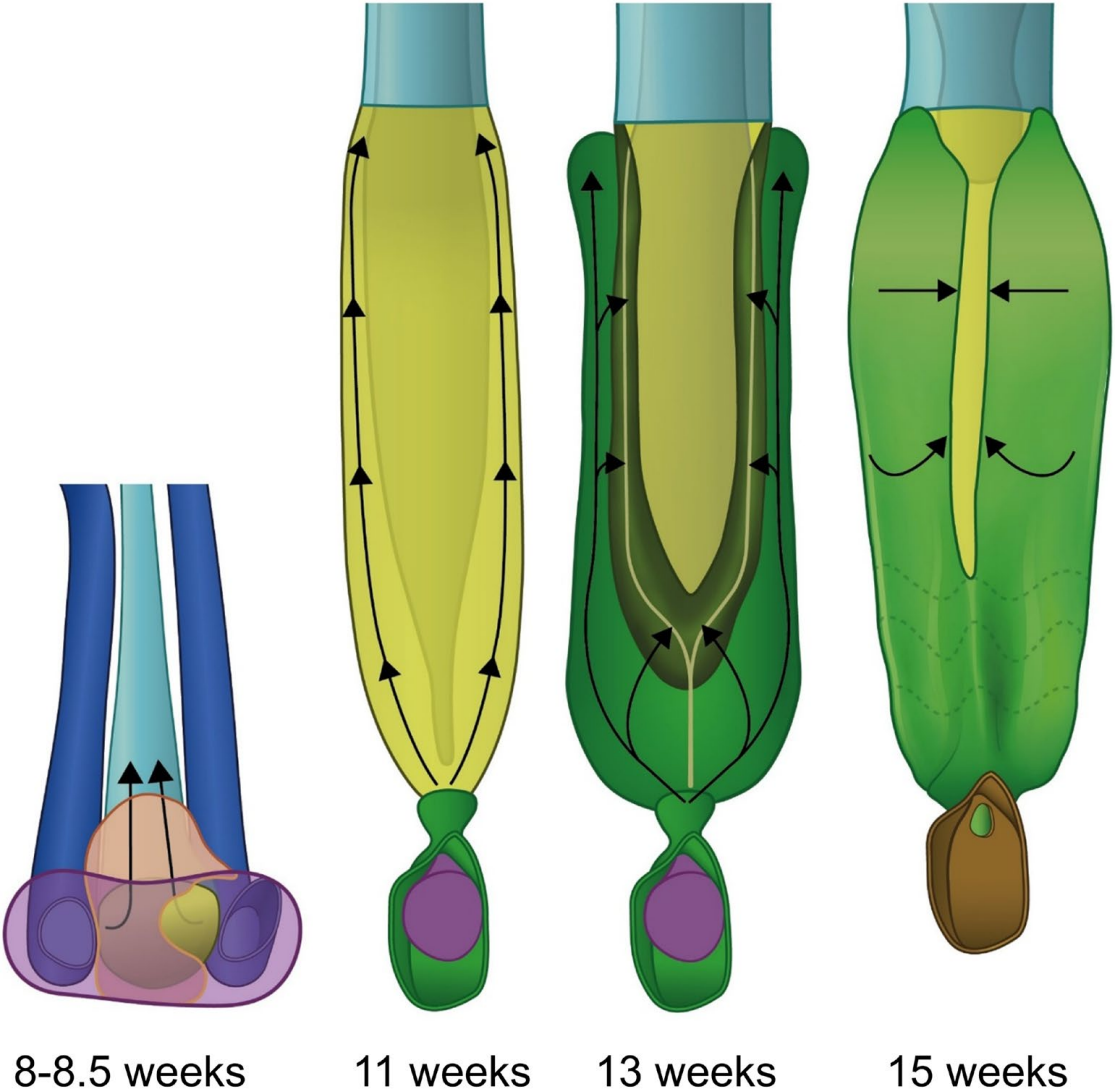
Proposed two‐phase model for the infiltration of urogenital‐sinus cells into the Müllerian epithelium of the uterovaginal canal, and the subsequent elimination of the Müllerian epithelial cells. We have named the epithelium with a fragmented basement membrane, regressive cylindrical Müllerian cells, and the presence of small urogenital epithelial cells “transformational epithelium,” and the subsequent elimination of the Müllerian epithelial cells the “purging process.” At ~8.5 weeks, the urogenital epithelium overlying the Müllerian hill differentiates (purple), infiltrates the underlying Müllerian epithelium (cyan), and establishes an area with transformational epithelium (yellow). Note that a dense mesenchymal wedge (orang) temporarily prevents direct contact between both epithelia on the right side. Subsequently, the urogenital cells differentiate further into tall apical cells with a “clear” cytoplasm (purple), whereas the basal cells retain their small‐cell phenotype (green; so‐called “sinusal” cells). Cranial expansion of the sinusal cells (arrows) continues until they have reached the uterovaginal border at ~11 weeks, where they stop infiltrating the uterovaginal epithelium. Instead, the sinusal cells now produce so‐called “vaginal plates” of purely sinusal cells on the lateral and dorsal sides of the transformational epithelium. These vaginal plates expand medially (horizontal course of arrows at 13 weeks) as a result of the selective removal of the Müllerian epithelial cells from the transformational epithelium (dark‐green ridge between the “pure” sinusal epithelium (green) and the remaining transformational epithelium (yellow)). The replacement of Müllerian by urogenital epithelium is complete when the purging zones have reached the midline (~16 weeks). The 15‐week model shows the caudo‐cranial gradient in the completion of this process.

## 5 Growth

We observed two phases of exponential growth of the uterovaginal canal (Figure 11). The early growth phase was observed between 7 and 10 weeks of development, is present throughout the uterovaginal canal, and corresponds with the descent of the Müllerian tubercle to the vaginal vestibule (Figure 11; Koff 1933). The second growth phase, with a ~twofold slower growth rate, starts at 9–11 weeks of development and is similar (Figure 11) or more prominent (Koff 1933) in the vagina than in the uterus. The growth rate of an embryological structure usually declines when differentiation starts. If this generalization also applies to the vaginal epithelium, the appearance of the vaginal plates and the finalization of the removal of Müllerian epithelial cells are two landmarks in vaginal development.

Our findings that the configuration of the lateral and medial vaginal cell plates changes from orthogonal to a zigzag or “W” arrangement reveal the location of a lateral growth node, while the passage of the caudal part of the vagina through the pelvic floor (figure 14 in Hülsman, Köhler, et al. 2025a) underscores its net caudal extension. Our histological data indicate that the growth and the secondary branching pattern of the vaginal plates play a prominent role in this growth. For this reason, Bulmer bestowed the dynamic, but unfortunately rather non‐descriptive term “upgrowth” to this caudal part of the vagina (Bulmer 1957).

## 6 The Lining of the Vaginal Canal

After 16–19 weeks of development, the sinusal epithelial cells have replaced the Müllerian epithelial cells in the vagina (Figure 12; Meyer 1938, Bulmer 1957, Robboy et al. 2017). A majority of the investigators who have studied vaginal development underwrite this conclusion (Vilas 1932; Meyer 1934, 1936, 1937; Kempermann 1935; Bulmer 1957). Kempermann is special because he revised his position with respect to the origin of the vaginal epithelium from Wolffian‐duct cells (Kempermann 1931) to urogenital‐sinus cells (Kempermann 1935). In contrast, Matejka, who provided a detailed and accurate account of the morphogenesis of the vagina (Matejka 1959), denied the epithelial remodeling of the vaginal wall altogether. In our view, the underlying reason for much of the discussion about the origin of the definitive vaginal epithelium is the close topographic association of the caudal parts of the Wolffian and Müllerian in 9–10‐week‐old fetuses and the lineage attribution of the epithelial cells that line the widened caudal ends of the Wolffian ducts.

Between 8 and 10 weeks of development, the uterovaginal canal is, apart from a markedly thicker epithelium at its caudal end, a fairly uniform tube. This caudal “head” is flanked by the wide caudal parts of the Wolffian ducts (Figures 3 and 4), but both epithelia remain separated by a well‐developed basement membrane. The acute and strikingly local regression of the Wolffian‐duct epithelium that contacts the clear cells may well point at a role for the clear cells in neutralizing a potentially adverse effect of the Wolffian epithelial cells on the Müllerian epithelium. The proponents of the Wolffian‐duct epithelium as a source of the definitive epithelium of the vagina (e.g., Mijsberg 1924 and [temporarily] Kempermann 1931) seem to have missed this prominent feature. At ~11 weeks of development, cell detritus and casts of dead cells, presumably of Wolffian‐duct origin, appear in the caudal lumens of the Wolffian ducts (Figure 4) but, as first reported by Kempermann (1935), the epithelium of their walls is replaced by healthy‐looking sinusal clear cells (this study; figure 2 in Vilas 1932). In 1–2‐week‐older fetuses, the Wolffian ducts still drain just laterally of the tip of the sinusal funnel (Figures 6 and S2). These findings strongly argue against the hypothesis that the Wolffian outlets migrate cranially to join the lower end of the vagina rather than the urogenital sinus (Meyer 1934, 1936, 1937; Mijsberg 1924; Koff 1933; Kempermann 1931). Indeed, Kempermann changed his hypothesis of the origin of the vaginal epithelium from Wolffian to sinusal when he realized that the casts of dead Wolffian cells are located in a part of the duct that is lined by healthy sinusal clear cells (Kempermann 1935).

Kempermann first described the so‐called “sinovaginal bulbs” (“Bulbus des Sinus urogenitalis”; his Figures 4 and 7; Kempermann 1931) as structures that correspond to what we have described as the large wings of the butterfly configuration of the urogenital sinus at the level of the tubercle (Figures 3 and 4). According to Drews (Bok and Drews 1983; Drews 2007), Koff misinterpreted for the same reason as Kempermann (Kempermann 1931) the caudally wide Wolffian ducts as bilateral epithelial protrusions of the dorsal wall of the urogenital sinus (“posterior evaginations”) and, hence, named them “sinovaginal bulbs” (Koff 1933). This “error” may have to be attributed to his aversion to histology (he preferred the analysis of reconstructions over that of sections; p. 63 in Koff 1933). Koff then wrote that these protrusions are continuous with the solid tip (equivalent to our “head”) of the uterovaginal canal and the Wolffian ducts. In his opinion, the epithelia of these bulbs “coalesce” with the solid tip of the uterovaginal canal to form the vaginal plates (p. 70 in Koff 1933). This misinterpretation probably arose because the vaginal plates extend from the urogenital sinus just caudal to the Wolffian outlets (Figures 6 and S2). Koff's sinovaginal bulbs seem to be identical to Bulmer's (Plate I, panels 4–6) and Vilas' two dorsolateral projections and single dorsomedial projection that fuse at ~13 weeks to form the single “Sinus‐Epithel Platte” (Vilas 1932) or “sinus upgrowth” (Bulmer 1957). The dorsolateral and dorsomedial projections represent just another description of our lateral and medial folds (Figures 6 and 8). However, in our opinion, these folds do not fuse, but develop inside the genital cord as surface‐extending folds of the epithelium that, at 12 and 13 weeks, is developing a supportive surrounding fascia. This surface extension is necessary to extend the caudal vaginal lumen to a similar diameter as the cranial vaginal lumen. Drews states that the caudal segments of the Wolffian ducts are incorporated into the vaginal plates (Bok and Drews 1983), whereas we report, instead, that the vaginal plates originate from sinusal epithelial cells that penetrate the genital cord (Figures 6 and S2). As stated above, Robboy's immunohistochemical findings (Robboy et al. 2017) reject Drews' interpretation. The reason for the discrepant interpretations is probably again the very close topographic relation of the Wolffian‐duct outlets and the forming lateral vaginal plates just caudal to the Wolffian‐duct outlets (Figures 6 and S2). An interesting contemporary, but acerbic assessment of the older accounts that we discussed can be found in (von Lippmann 1939).

A frequently raised issue is why the replacement of Müllerian epithelium by sinusal (Koff 1933) or Wolffian epithelium (Mijsberg 1924) would occur only in the caudal ~20% of the vagina. According to Bulmer (Bulmer 1957), the caudocranial direction of differentiation in the vaginal epithelium and the resulting lesser degree of differentiation of the cranial epithelial cells of the vagina is the underlying cause. We propose that both Mijsberg and Koff considered only the head of the uterovaginal canal (~20% of the craniocaudal length of the vagina) as undergoing epithelial remodeling, because only this part developed an elaborate system of sinusal folds and dilations (Figure 8). We established, however, that the epithelium surrounding the cranially wide lumen of the Müllerian duct was, although ~40% thinner than the caudal equivalent, of the transformational type.

## 7 Species Differences

As mentioned above, studies that specifically stained cells of Müllerian or urogenital origin in human fetal urogenital tracts demonstrated that the epithelium of the entire vagina is eventually of urogenital origin (Robboy et al. 2017). Differential genetic labeling of the epithelial cells of Müllerian *plus* Wolffian, Wolffian, or urogenital origin similarly revealed that the caudal, non‐luminal part of the perinatal murine vagina is made up of urogenital epithelial cells (Kurita 2010). This finding suggests that late prenatal development of the vaginal epithelium in mice is comparable to early fetal development in humans. Four weeks after birth, however, the contribution of urogenital‐sinus epithelium to the vaginal epithelium is reduced to a few Müllerian cells on the vestibular side of the hymen in mice (figure 2C in Kurita 2011). Apparently, the local vaginal environment in the mouse changes postnatally to increase the growth of the caudal Müllerian epithelium at the expense of the urogenital epithelium. The caudal, non‐luminal part of the vagina behaves as a segment and could, therefore, be determined by an attenuation of the expression of caudal Hox genes. Hox13 is the Hox gene expressed in the vagina and is sensitive to variations in ambient androgen concentrations (Norris et al. 2009). We have proposed an activation of the HoxA‐10 gene to explain the development of the uterine horns in rodents (Hülsman, Köhler, et al. 2025a). In rodents, postnatal downregulation of Hox13 by, for example, androgens (Norris et al. 2009) may be responsible for the change in epithelial phenotype of the caudal‐most part of the vagina.

## 8 Conclusion

We report that the fetal vaginal epithelium changes from Müllerian to sinusal in origin and propose a mechanistic model for the process. Our study showed the key importance of a detailed analysis of the histological architecture of the epithelia involved. In addition, it emphasized that a sufficient number of high‐resolution microscopical images is as necessary for a proper characterization of tissues and structures as a well‐formulated description. If such complementary sources of information are not provided in the available literature, as was experienced in the present study, it takes a time‐consuming effort to understand supposedly signature terms like “sinovaginal bulbs,” “dorsolateral projections,” and “upgrowths.”

## Supporting information


**Figure S1:** Histological sections of the bifid caudal tip of the uterovaginal canal at 8 weeks. These sections correspond to panels (B)–(E) of Figure 1, except that the contours were omitted to be able to study the epithelia. All panels have the same magnification. Bar = 0.2 mm.

**Figure S2:** Serial sections of the topographic relation of the Wolffian ducts and the lateral vaginal plates at 12 weeks. Panels (A)–(D) are cranial to caudal sections near the tip of the sinusal funnel. Clear cells are present in the funnel lumen. The Wolffian duct has a distinctive columnar epithelium and contacts the sinusal funnel dorsolaterally. The vaginal plates are identifiable by their strongly staining cytoplasm and tête‐à‐tête arrangement of the epithelium. Panels (A) and (D) correspond to panels (F) and (A) of Figure 8. All panels have the same magnification. Bar = 0.1 mm.

**Figure S3:** The sinusal funnel ends separately from the urethra on the vaginal vestibule at 13 weeks. Panel (A) is a sagittal midline section of the uterovaginal canal entering the vaginal vestibule. Panel (B) is a close‐up of the caudal part in which clear cells, vaginal plate and the purging zone is present. CC: clear cells; PZ: purging zone; VP: vaginal plate. Bars: 0.5 mm in panel (A) and 0.1 mm in panel (B).

**Figure S4:** Transverse sections through the uterovaginal canal at 15 weeks. Starting caudally (panel A) a branching sinusal epithelium with two dorsal and one ventral vaginal plate is present. More cranially (panels B–D) the epithelium branches less and gradually become a flat plane (C). Panel (B1) reveals the presence of the last non‐epithelial cells in the midline between both vaginal plates. The uterovaginal canal is the smallest at the level of the cervix (panel D). More cranially the lumen of the uterus has a wavy pattern (E). At the most cranial point of the uterovaginal canal, the genital cord transforms into the tissue cuff of the uterine tubes (F). Bars: 0.5 mm.
**3D‐PDF instructions.** To view the interactive 3D‐PDFs in their full potential you need to download the 3D‐PDFs to your computer (a 3D‐PDF can be opened on any computer as long as it contains the Adobe PDF reader). To activate the 3D‐PDF you need to click on the model. A toolbar appears at the top of the screen. Under options, you must state that you trust this document. If you then click on the model (version 24 and later: with the right mouse button), a toolbar appears on the left side of your screen (on the right side in version 24 and later) that includes the option “model tree.” The model tree displays a list of structures in the upper box and preset viewing options in the lower box. The list of visible structures can be modified by marking or unmarking a structure. We advise to start with a basal configuration that contains a few structures only and add structures to this simple configuration rather than the other way around: “dress, do not undress.” To manipulate the reconstruction, press the left mouse button to rotate it, the scroll button to zoom in or out, and the left and right mouse buttons simultaneously to move the embryo across the screen. The color code is identical in all figures, and all structures are listed by the same name and relative position in the “model tree.” The edges of the scale cube are 1 mm.

**Figure S5:** Interactive 3D‐pdf of the urogenital region of a ~8.5‐week‐old human embryo (EYO295).

**Figure S6:** Interactive 3D‐pdf of the urogenital region of a ~14‐week‐old human embryo (ME29).

**Figure S7:** Interactive 3D‐pdf of the urogenital region of a ~20‐week‐old human embryo (S2290).

## Data Availability

The data that support the findings of this study are available from the corresponding author upon reasonable request.
