# Supplementary figures and images for: The Development of the Human Female Reproductive Tract: Part 2—Vagina
Source: Clin Anat. 2025 Aug 24;39(1):112–31. doi: 10.1002/ca.70015 (PMC12747650; doi:10.1002/ca.70015)

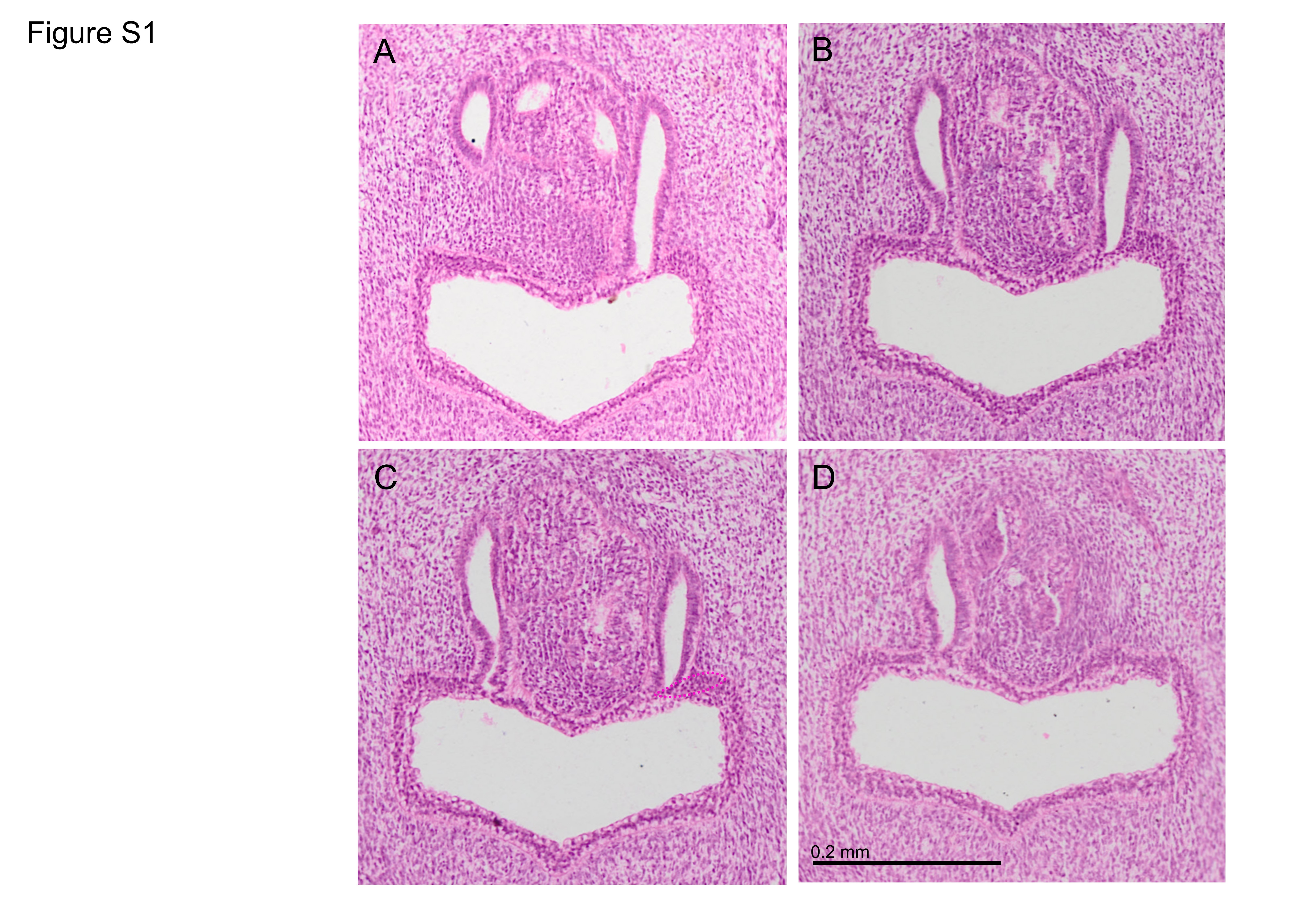

Supplement: Supplementary file 1 — Figure S1: Histological sections of the bifid caudal tip of the uterovaginal canal at 8 weeks. These sections correspond to panels (B)–(E) of Figure 1, except that the contours were omitted to be able to study the epithelia. All panels have the same magnification. Bar = 0.2 mm. [file CA-39-112-s001.jpg]

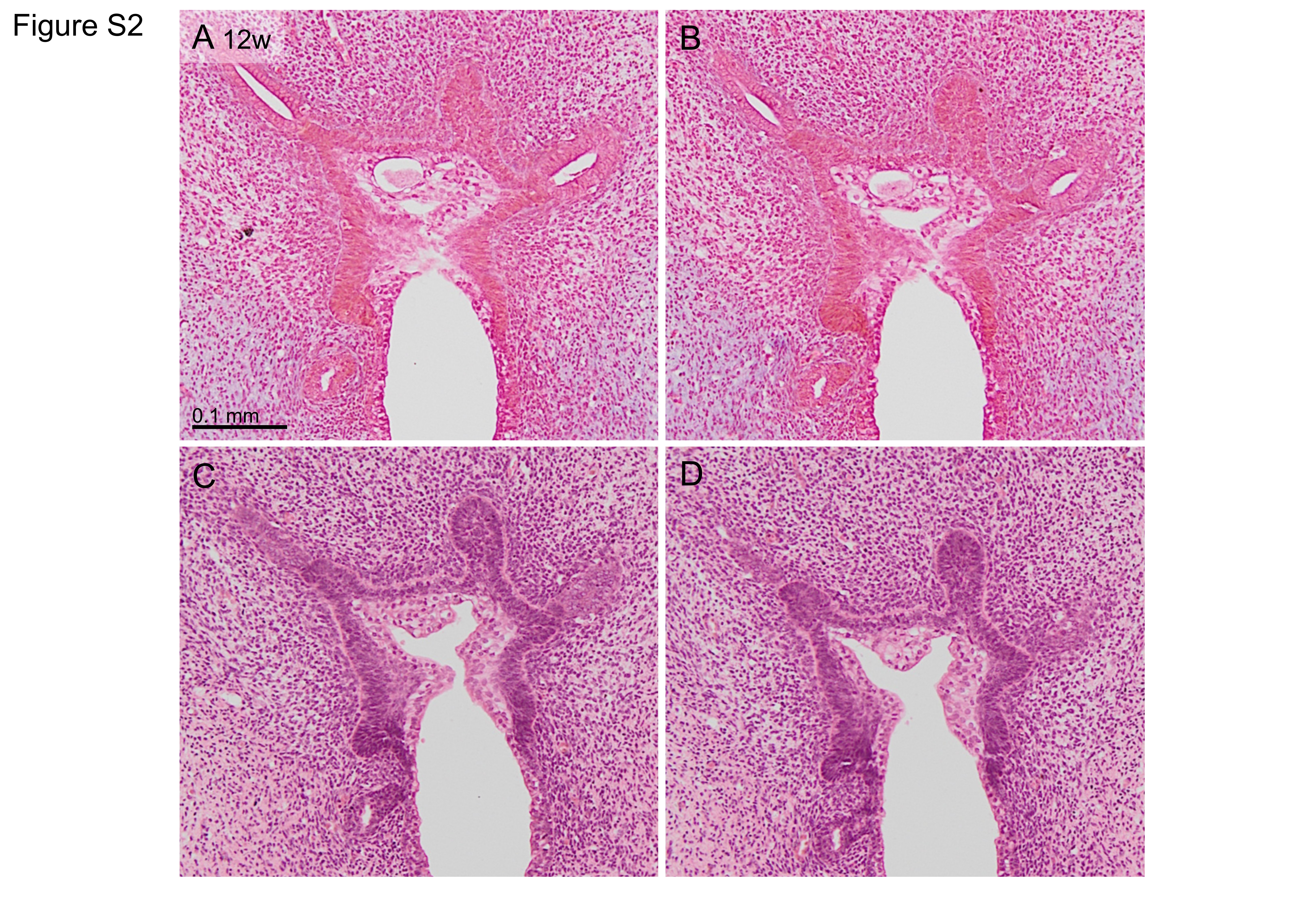

Supplement: Supplementary file 2 — Figure S2: Serial sections of the topographic relation of the Wolffian ducts and the lateral vaginal plates at 12 weeks. Panels (A)–(D) are cranial to caudal sections near the tip of the sinusal funnel. Clear cells are present in the funnel lumen. The Wolffian duct has a distinctive columnar epithelium and contacts the sinusal funnel dorsolaterally. The vaginal plates are identifiable by their strongly staining cytoplasm and tête‐à‐tête arrangement of the epithelium. Panels (A) and (D) correspond to panels (F) and (A) of Figure 8. All panels have the same magnification. Bar = 0.1 mm. [file CA-39-112-s002.jpg]

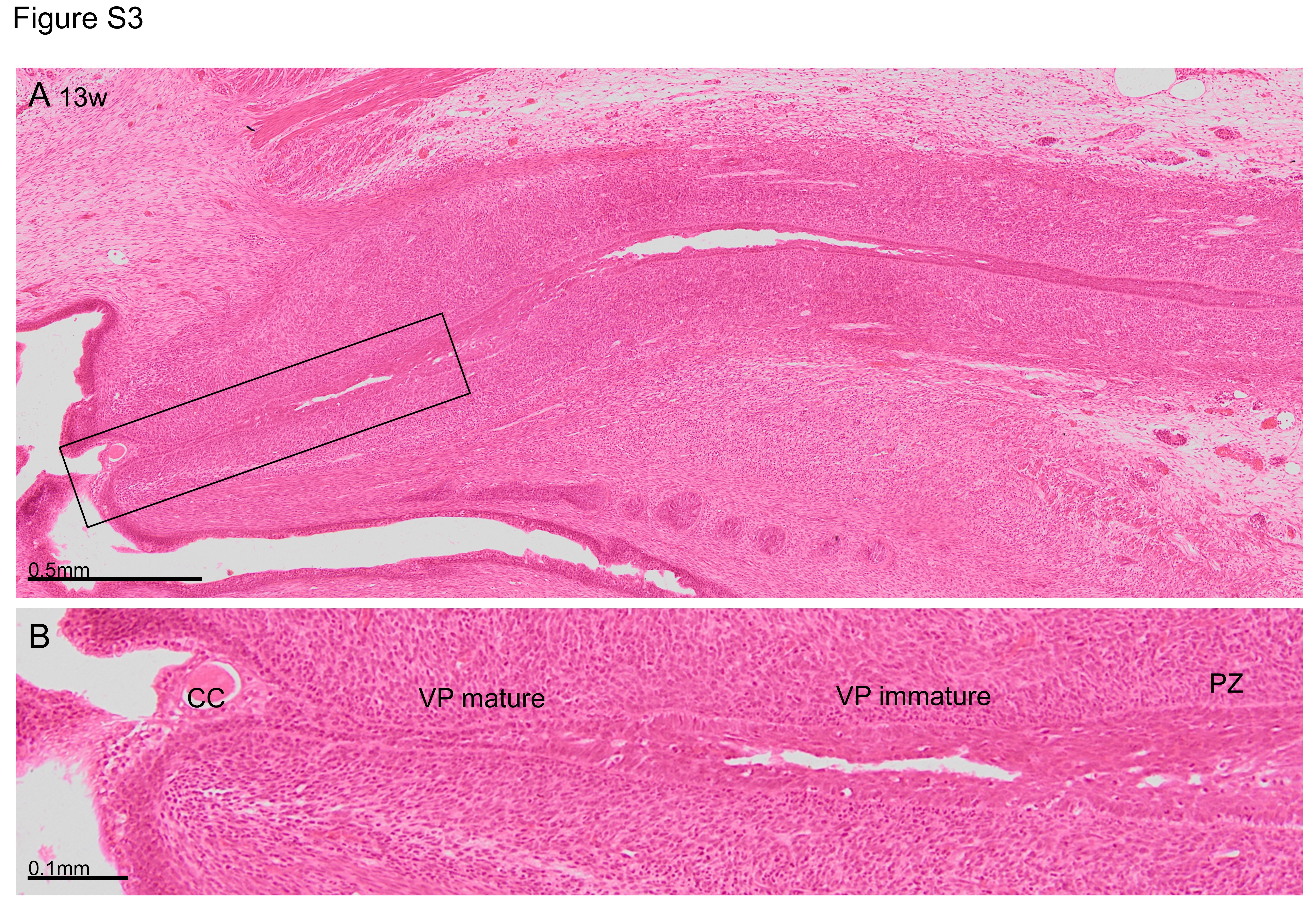

Supplement: Supplementary file 3 — Figure S3: The sinusal funnel ends separately from the urethra on the vaginal vestibule at 13 weeks. Panel (A) is a sagittal midline section of the uterovaginal canal entering the vaginal vestibule. Panel (B) is a close‐up of the caudal part in which clear cells, vaginal plate and the purging zone is present. CC: clear cells; PZ: purging zone; VP: vaginal plate. Bars: 0.5 mm in panel (A) and 0.1 mm in panel (B). [file CA-39-112-s004.jpg]

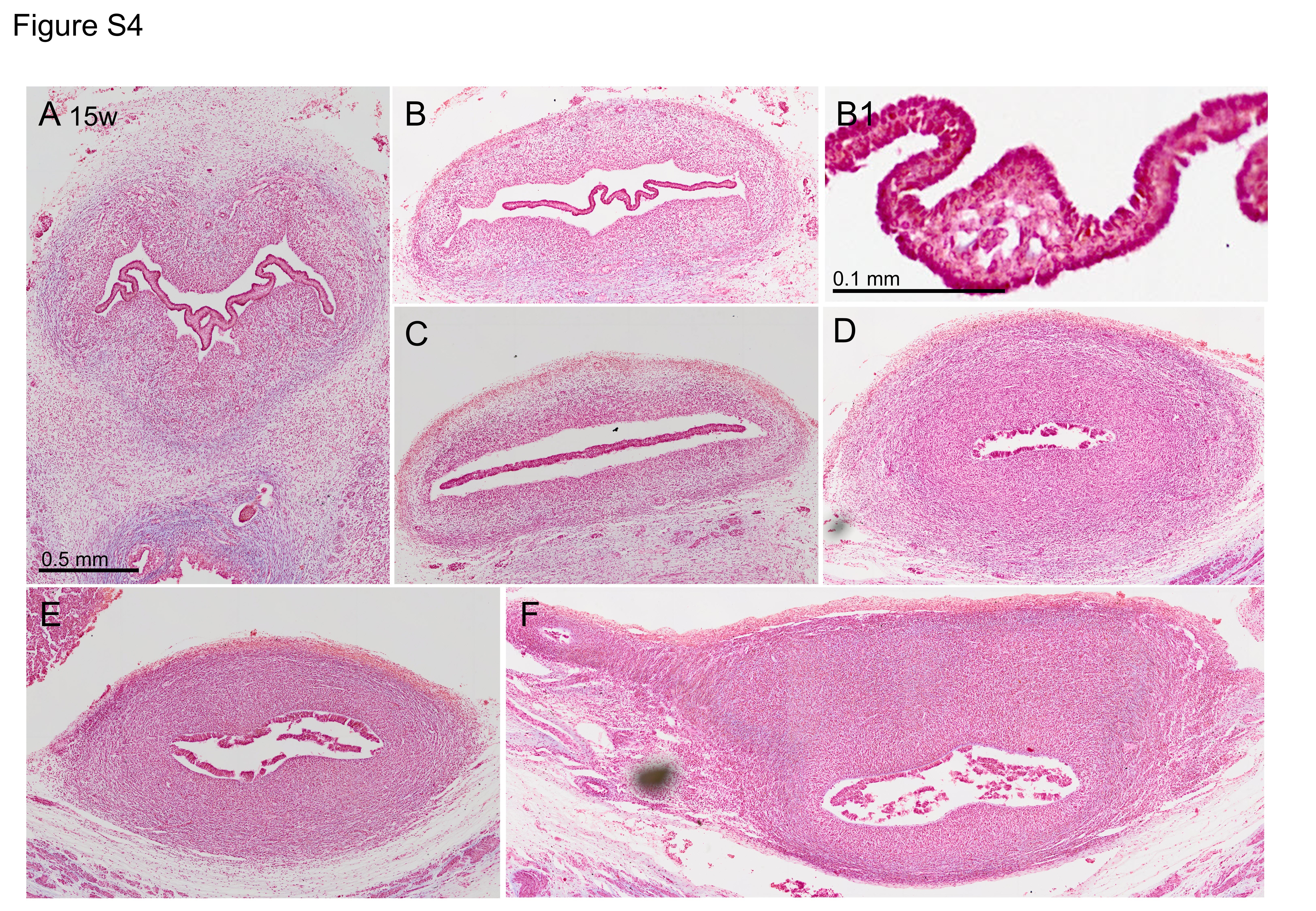

Supplement: Supplementary file 4 — Figure S4: Transverse sections through the uterovaginal canal at 15 weeks. Starting caudally (panel A) a branching sinusal epithelium with two dorsal and one ventral vaginal plate is present. More cranially (panels B–D) the epithelium branches less and gradually become a flat plane (C). Panel (B1) reveals the presence of the last non‐epithelial cells in the midline between both vaginal plates. The uterovaginal canal is the smallest at the level of the cervix (panel D). More cranially the lumen of the uterus has a wavy pattern (E). At the most cranial point of the uterovaginal canal, the genital cord transforms into the tissue cuff of the uterine tubes (F). Bars: 0.5 mm. 3D‐PDF instructions. To view the interactive 3D‐PDFs in their full potential you need to download the 3D‐PDFs to your computer (a 3D‐PDF can be opened on any computer as long as it contains the Adobe PDF reader). To activate the 3D‐PDF you need to click on the model. A toolbar appears at the top of the screen. Under options, you must state that you trust this document. If you then click on the model (version 24 and later: with the right mouse button), a toolbar appears on the left side of your screen (on the right side in version 24 and later) that includes the option “model tree.” The model tree displays a list of structures in the upper box and preset viewing options in the lower box. The list of visible structures can be modified by marking or unmarking a structure. We advise to start with a basal configuration that contains a few structures only and add structures to this simple configuration rather than the other way around: “dress, do not undress.” To manipulate the reconstruction, press the left mouse button to rotate it, the scroll button to zoom in or out, and the left and right mouse buttons simultaneously to move the embryo across the screen. The color code is identical in all figures, and all structures are listed by the same name and relative position in the “model tree.” The edges of th [file CA-39-112-s007.jpg]

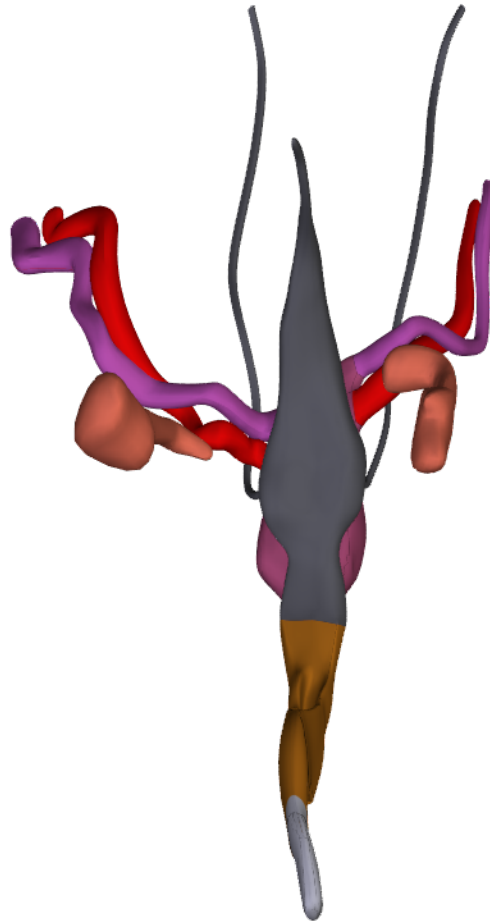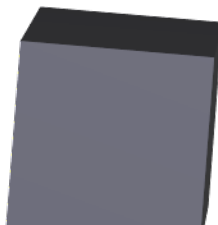

Supplement: Supplementary file 5 — Figure S5: Interactive 3D‐pdf of the urogenital region of a ~8.5‐week‐old human embryo (EYO295). [file CA-39-112-s006.pdf]

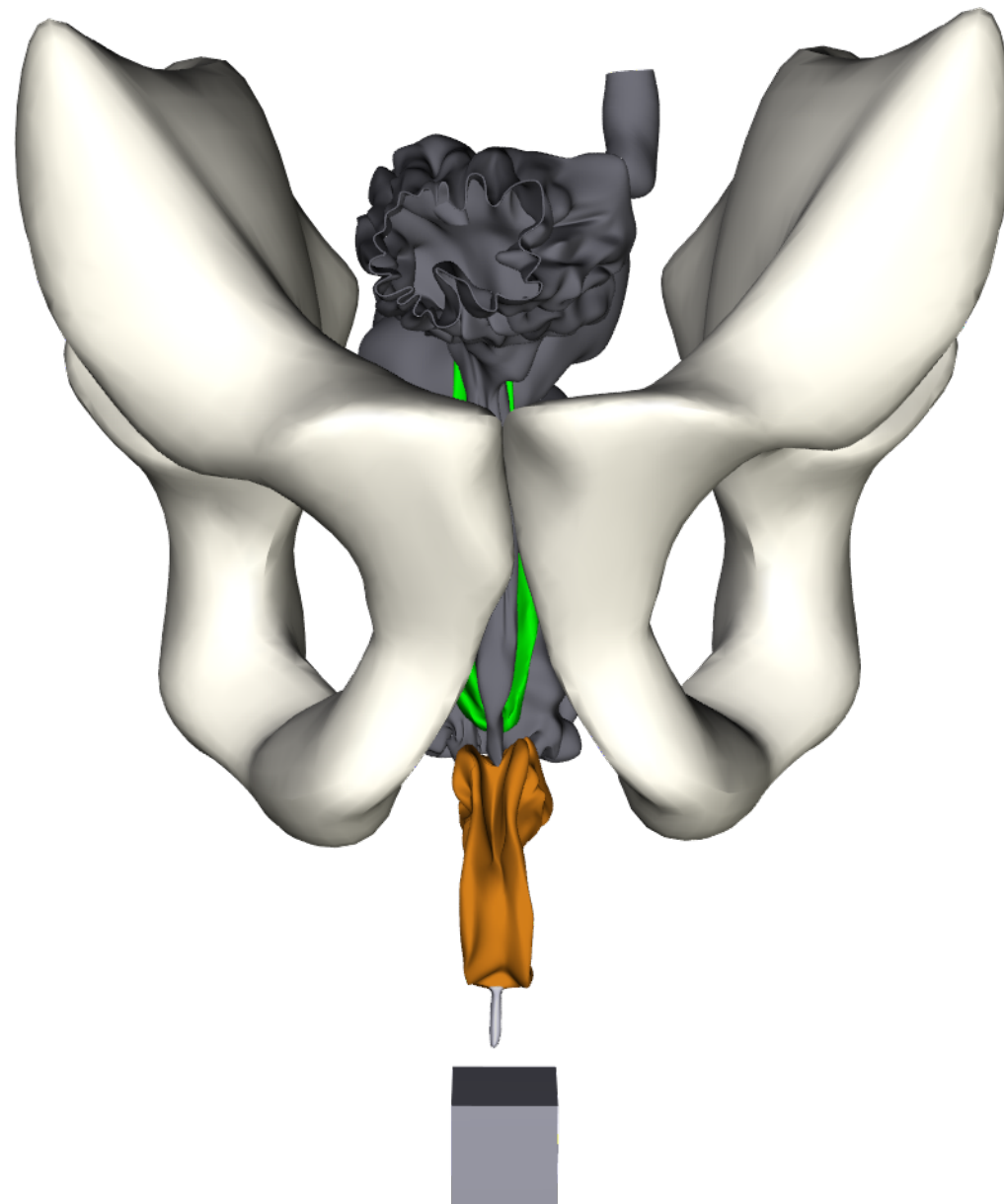

Supplement: Supplementary file 6 — Figure S6: Interactive 3D‐pdf of the urogenital region of a ~14‐week‐old human embryo (ME29). [file CA-39-112-s005.pdf]

20 Weeks (S2290)

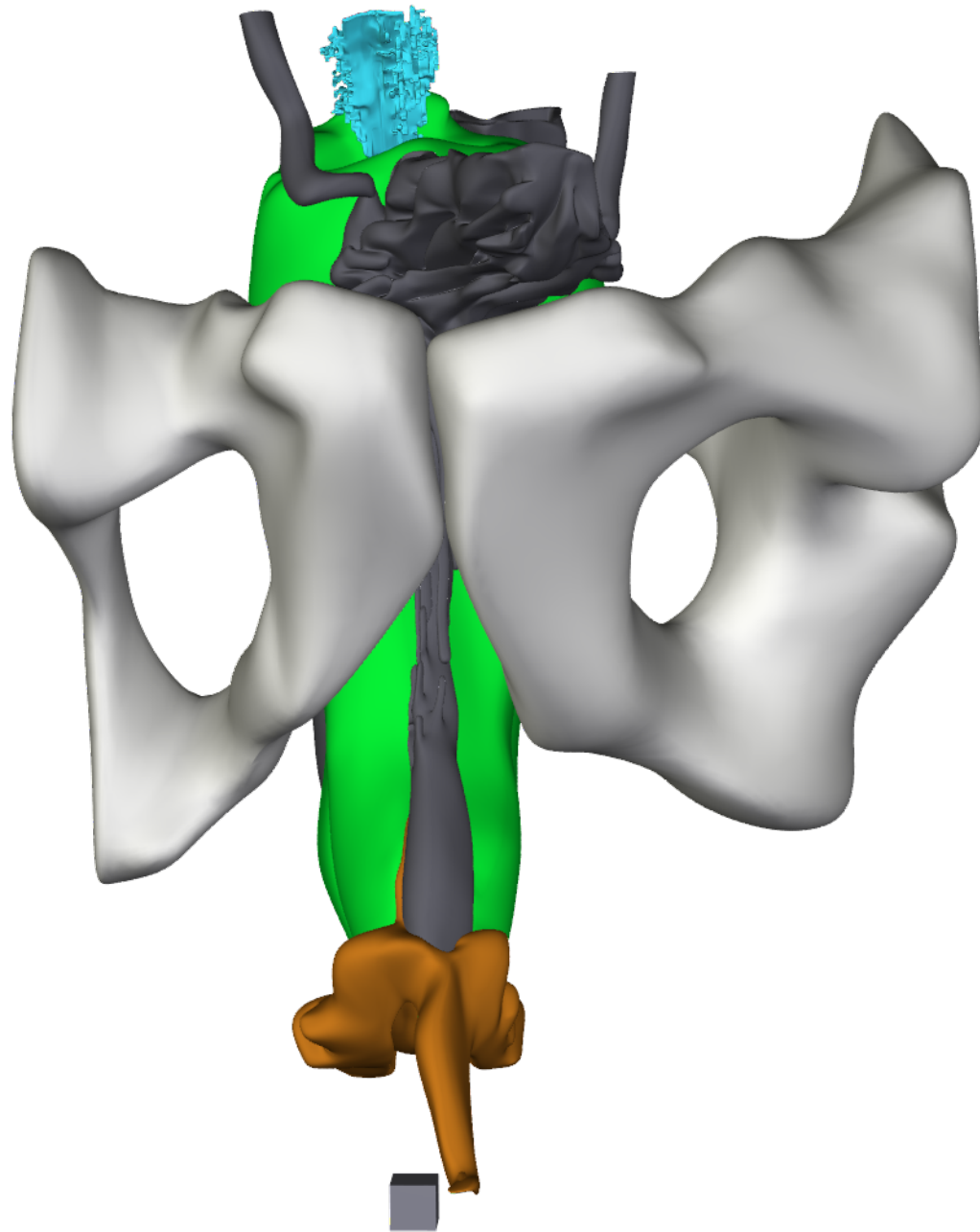

Supplement: Supplementary file 7 — Figure S7: Interactive 3D‐pdf of the urogenital region of a ~20‐week‐old human embryo (S2290). [file CA-39-112-s003.pdf]
